# Pooled two-cohort MRI body composition phenotyping with open-source deep learning

**DOI:** 10.1038/s43856-026-01888-w

**Published:** 2026-08-29

**Authors:** Christian J. Mertens, Hartmut Häntze, Sebastian Ziegelmayer, Jakob Nikolas Kather, Daniel Truhn, Su Hwan Kim, Felix Busch, Dominik Weller, Benedikt Wiestler, Markus Graf, Fabian Bamberg, Christopher L. Schlett, Jakob B. Weiss, Steffen Ringhof, Elif Can, Jeanette Schulz-Menger, Thoralf Niendorf, Jacqueline Lammert, Isabel Molwitz, Avan Kader, Alessa Hering, Aymen Meddeb, Jawed Nawabi, Matthias B. Schulze, Thomas Keil, Stefan N. Willich, Lilian Krist, Martin Hadamitzky, Anke Hannemann, Florian Bassermann, Daniel Rueckert, Tobias Pischon, Alexander Hapfelmeier, Marcus R. Makowski, Keno K. Bressem, Lisa C. Adams

**Affiliations:** 1 Institute for Diagnostic and Interventional Radiology, School of Medicine and Health, TUM Klinikum, Technical University of Munich (TUM), Munich, Germany; 2 Diagnostic Image Analysis Group, Department of Medical Imaging, Radboud University Medical Center, Nijmegen, The Netherlands; 3 Else Kroener Fresenius Center for Digital Health, Dresden University of Technology, Dresden, Germany; 4 Department of Medicine I, University Hospital Dresden, Dresden, Germany; 5 Medical Oncology, National Center for Tumour Diseases (NCT), University Hospital Heidelberg, Heidelberg, Germany; 6 Department for Diagnostic and Interventional Radiology, University Hospital Aachen, Aachen, Germany; 7 Department of Diagnostic and Interventional Neuroradiology, School of Medicine and Health, TUM Klinikum Rechts der Isar, Technical University of Munich, Munich, Germany; 8 Department of Diagnostic and Interventional Radiology, Medical Center, University of Freiburg, Faculty of Medicine, University of Freiburg, Freiburg im Breisgau, Germany; 9 Charité, Universitätsmedizin Berlin, Corporate Member of Freie Universität Berlin and Humboldt-Universität zu Berlin, ECRC Experimental and Clinical Research Center, Berlin, Germany; 10 Working Group on CMR, Experimental and Clinical Research Center, a Joint Cooperation Between the Max-Delbrück-Center for Molecular Medicine and the Charité, Universitätsmedizin Berlin, Berlin, Germany; 11 DZHK (German Centre for Cardiovascular Research), Berlin, Germany; 12 Department of Cardiology and Nephrology, Helios Hospital Berlin-Buch, Berlin, Germany; 13 Max-Delbrück-Center for Molecular Medicine in the Helmholtz Association (MDC), Berlin Ultrahigh Field Facility (B.U.F.F.), Berlin, Germany; 14 Experimental and Clinical Research Center (ECRC), Charité-Universitätsmedizin Berlin, A Joint Cooperation Between the Charité Medical Faculty and the Max-Delbrück Center for Molecular Medicine in the Helmholtz Association, Berlin, Germany; 15 Department of Gynecology and Center for Hereditary Breast and Ovarian Cancer, Technical University of Munich (TUM), School of Medicine and Health, Klinikum rechts der Isar, TUM University Hospital, Munich, Germany; 16 Department of Diagnostic and Interventional Radiology and Nuclear Medicine, University Medical Center Hamburg-Eppendorf, Hamburg, Germany; 17 Department of Neuroradiology, Charité Universitätsmedizin Berlin, Berlin, Germany; 18 Department of Molecular Epidemiology, German Institute of Human Nutrition Potsdam-Rehbrücke, Nuthetal, Germany; 19 Institute of Nutrition Science, University of Potsdam, Nuthetal, Germany; 20 Institute of Social Medicine, Epidemiology and Health Economics, Charité, Universitätsmedizin Berlin, Corporate Member of Freie Universität Berlin and Humboldt-Universität zu Berlin, Berlin, Germany; 21 Department of Cardiovascular Radiology and Nuclear Medicine, German Heart Center Munich, TUM University Hospital, Munich, Germany; 22 Institute of Clinical Chemistry and Laboratory Medicine, University Medicine Greifswald, Greifswald, Germany; 23 Department of Medicine III, Technical University of Munich, TUM School of Medicine and Health, Munich, Germany; 24 Center for Translational Cancer Research (TranslaTUM), Technical University of Munich, TUM School of Medicine and Health, Munich, Germany; 25 Munich Center for Machine Learning (MCML), Munich, Germany; 26 Chair of AI in Healthcare and Medicine, Technical University of Munich (TUM) and TUM University Hospital, Munich, Germany; 27 Department of Computing, Imperial College London, London, UK; 28 Molecular Epidemiology Research Group, Max-Delbrück-Center for Molecular Medicine in the Helmholtz Association (MDC), Berlin, Germany; 29 Max-Delbrück-Center for Molecular Medicine in the Helmholtz Association (MDC), Biobank Technology Platform, Berlin, Germany; 30 Charité, Universitätsmedizin Berlin, Corporate Member of Freie Universität Berlin and Humboldt-Universität zu Berlin, Berlin, Germany; 31 Technische Universität of Munich, School of Medicine and Health, TUM University Hospital Rechts der Isar, Institute of AI and Informatics in Medicine and Institute of General Practice and Health Services Research, Munich, Germany

**Keywords:** Diagnostic markers, Cardiovascular diseases

## Abstract

**Background:**

Body mass index fails to capture variation in fat and muscle distribution that determines metabolic health and disease risk. MRI enables radiation-free quantification of regional body composition, yet scalable open-source tools applied in pooled cohorts with differing acquisition protocols have been lacking.

**Methods:**

MRSegmentator, an open-source nnU-Net-based pipeline, was applied to quantify visceral adipose tissue (VAT), abdominal subcutaneous adipose tissue (ASAT), gluteofemoral adipose tissue (GFAT), trunk musculature, and the liver mask used for liver fat-fraction estimation in 45,851 adults from the German National Cohort (*n* = 26,877, 3 T multi-centre Siemens) and UK Biobank (*n* = 18,974, 1.5 T Siemens). Population-scale compartment volumes were segmented from stitched in-phase gradient-echo (GRE) images in both cohorts; liver fat fraction was calculated from fat-only and water-only images. The annotated development data comprised NAKO T2-HASTE and UKB Dixon reconstructions. A single pooled model was applied without site-specific adaptation. A separate two-reader agreement study used 50 scans from these annotated development-sequence domains. Associations between BMI-adjusted body composition and cardiometabolic conditions were estimated using generalized linear mixed-effects models. Incremental discrimination beyond age, BMI, and waist-to-hip ratio was assessed.

**Results:**

Five-fold participant-stratified internal cross-validation against curated human-in-the-loop development references comprising UKB Dixon and NAKO T2-HASTE yielded a mean Dice of 0.91. In a separate 50-scan reader study on these annotated development-sequence images, overall reader–reader Dice was 0.937 and overall algorithm–reader Dice was 0.908. The trained pipeline was then used to segment compartment volumes from stitched in-phase GRE inputs in both cohorts, while liver fat fraction was calculated from fat-only and water-only images; direct sequence-matched validation on NAKO GRE was not performed. VAT showed the strongest positive associations with cardiometabolic conditions, while GFAT showed inverse associations, most prominently for type 2 diabetes (OR 0.69, 95% CI 0.66 to 0.72). Disease-specific body-composition phenotypes were identified, with type 2 diabetes characterized by elevated VAT, reduced GFAT, and increased liver fat. MRI-derived compartments modestly improved discrimination for type 2 diabetes and hyperlipidemia beyond anthropometric measures.

**Conclusions:**

A single open-source deep-learning pipeline enabled pooled body-composition phenotyping in two cohorts and captured distributional variation in fat and muscle beyond BMI. High agreement in internal cross-validation (mean Dice 0.91) and the separate two-reader study support the annotated development-sequence analysis, while the population-scale application identified distinct disease-associated phenotypes and modest incremental discrimination beyond conventional anthropometry.

## Plain language summary

Body mass index (BMI), the standard measure for assessing obesity, cannot distinguish between different types of body fat or account for muscle mass. In this study, MRI scans of nearly 46,000 adults from Germany and the United Kingdom were analysed using a freely available computer program to measure regional fat deposits and trunk muscle volume automatically. Five-fold internal cross-validation on annotated UK Biobank Dixon and NAKO T2-HASTE development images yielded a mean Dice of 0.91. A separate comparison on 50 development-sequence scans found a reader–reader Dice score of 0.937 and a program–reader score of 0.908. The program was then used to segment compartment volumes from in-phase GRE images in both cohorts; liver fat was calculated from the fat-only and water-only images. People with more fat around internal organs had higher prevalence of heart disease and diabetes, while those with more fat around the hips had lower prevalence at the same BMI. Adding these MRI measurements to standard clinical information produced modest improvements in discrimination for diabetes and high cholesterol.

## Abstract (web summary)

Body mass index inadequately captures the distribution of fat and muscle, crucial for assessing metabolic health. Here, the authors introduce MRSegmentator, an open-source deep-learning tool, to analyze MRI data from large cohorts, revealing disease-specific body-composition phenotypes and enhancing discrimination of cardiometabolic conditions beyond traditional measures.

## Introduction

Body mass index (BMI) remains the dominant clinical metric for assessing obesity, yet it fails to capture variation in fat and muscle distribution that increasingly determines metabolic health and disease risk^1,2^. Individuals with identical BMI values can exhibit markedly different metabolic profiles depending on the relative distribution of visceral, subcutaneous, and gluteofemoral adipose tissue and skeletal muscle^2–6^. This heterogeneity limits BMI-based risk stratification and motivates imaging-based body composition phenotyping.

CT-based body composition analysis has validated this approach at the population scale. Automated pipelines applied to over 100,000 patients have demonstrated associations between regional fat and muscle measurements and cardiometabolic outcomes, including prediction of 10-year adverse events^7–10^. However, CT involves ionizing radiation, limiting its use in healthy screening populations and longitudinal monitoring. MRI provides a radiation-free alternative with superior soft tissue contrast, enabling differentiation of adipose tissue compartments, including gluteofemoral fat that is difficult to quantify on axial CT, and direct muscle volume quantification without attenuation-based surrogates^11–13^.

Despite these advantages, MRI-based body-composition analysis has lacked scalable open-source tools applied in pooled cohorts with differing acquisition protocols and field strengths. Prior large-scale MRI studies in the UK Biobank relied on 2D projections for fat-depot estimation or commercial platforms not available for independent validation^1,14^. Manual segmentation remains impractical at population scale, and sequence-matched validation across acquisition settings remains limited.

This study evaluates MRSegmentator, an open-source nnU-Net-based pipeline^15^. It was applied to 45,851 adults from the UK Biobank (*n* = 18,974) and the German National Cohort (*n* = 26,877)^16–18^. The primary aim was to apply a single pooled pipeline across two cohorts with different acquisition protocols, report segmentation performance on the available annotated UKB Dixon and NAKO T2-HASTE data, and contextualize this performance with two-reader agreement on the annotated sequence domains. Secondary aims were to determine whether the resulting measurements produced biologically plausible associations with cardiometabolic conditions in the pooled analysis, independent of BMI, and to characterize disease-specific body-composition phenotypes and incremental discriminative value beyond conventional anthropometric measures.

Here, we show high segmentation agreement in annotated UKB Dixon and NAKO T2-HASTE development data (mean Dice 0.91). The pooled model was then applied to stitched in-phase GRE inputs in both cohorts without site-specific adaptation; direct sequence-matched validation on NAKO GRE was not performed. VAT shows the strongest positive associations with cardiometabolic conditions, whereas GFAT shows the largest inverse association with type 2 diabetes (OR 0.69, 95% CI 0.66–0.72). Distinct body-composition phenotypes are identified for individual diseases. MRI-derived compartments modestly improve discrimination for type 2 diabetes and hyperlipidemia beyond age, BMI, and waist-to-hip ratio.

## Methods

This retrospective cross-sectional study was conducted under data access applications NAKO-836 and UKB-34479. The study protocol for the present analysis was approved by the Ethics Committee of the Technical University of Munich, School of Medicine and Health (Ethikkommission der Technischen Universität München; reference 2024-479-S-SB). Each cohort additionally holds its own ethics approval. The German National Cohort (NAKO) is a multi-centre study approved by the responsible ethics committees of the federal states of all participating study centres, with the lead vote provided by the Ethics Committee of the Bavarian State Chamber of Physicians (Ethikkommission der Bayerischen Landesärztekammer). The UK Biobank holds Research Tissue Bank approval from the North West Multi-centre Research Ethics Committee (REC reference 11/NW/0382), under which the present analysis was conducted without requiring separate study-specific ethics approval. All participants provided written informed consent. Complete protocols for both cohorts are available online at https://nako.de/wp-content/uploads/NAKO-Wissenschaftliches-Konzept.pdf for NAKO and https://www.ukbiobank.ac.uk/media/gnkeyh2q/study-rationale.pdf for UKB.

### Study cohorts

Data were obtained from the German National Cohort (NAKO) and UK Biobank (UKB) population-based imaging cohorts. NAKO enrolled 205,217 individuals aged 19–74 years at 18 centres in Germany^17,18^. Whole-body 3 T MRI was performed in 30,861 participants between 2014 and 2019 at five imaging centres using Magnetom Skyra scanners (Siemens Healthineers, Erlangen, Germany); MRI data from 30,397 participants were available for the present analysis. UKB is a prospective biomedical database of more than 500,000 adults aged 40–69 years^16^. MRI data and outcomes from 19,512 participants at two UKB centres were available, acquired on 1.5 T Siemens systems. The cohorts differ in field strength, scanner system, acquisition protocol, and participant demographics; these differences define the scope of the pooled application but do not constitute independent validation of equal performance.

For the population-scale body-composition analysis, in-phase gradient-echo (GRE) images were used in both cohorts; four contiguous NAKO partitions and six contiguous UKB partitions were stitched with an in-house tool. Participants were excluded if required GRE image types (in-phase, opposed-phase, fat-only, water-only) or expected partition numbers were missing, or if image dimensions were inconsistent (NAKO *n* = 358, UKB *n* = 91). Mean liver fat-fraction values > 0.6 indicated possible fat–water swap artefacts. Following visual verification, large-area swaps were excluded (NAKO *n* = 137, UKB  = 257), while localized swaps were masked before liver fat-fraction estimation. Additional exclusions were applied for missing anthropometric (NAKO *n* = 1, UKB *n* = 0), disease-status (NAKO *n* = 787), or health-behaviour data (NAKO *n* = 2237, UKB *n* = 190). The final datasets comprised 26,877 participants (14,969 men, 11,908 women) for NAKO and 18,974 (9094 men, 9880 women) for UKB. Participant selection and exclusions are detailed in Suppl. Fig. S1.

### Deep learning-based segmentation

Body-composition parameters were extracted using MRSegmentator^15^. This open-source pipeline is based on nnU-Net^19^. The 10-class MRI body-composition task produced subcutaneous and visceral fat, left and right rectus abdominis, oblique, and quadratus lumborum muscles, abdominal subcutaneous fat, and gluteofemoral fat; bilateral muscle classes were aggregated for analysis. The standard 40-class anatomical task supplied the liver mask used for liver fat-fraction estimation. Thus, the body-composition outputs are an additional task rather than replacements for the standard anatomical classes. The body-composition task was trained exclusively on UKB and NAKO data, whereas the anatomical task used to generate the liver mask incorporated training data beyond these cohorts^15^. ASAT was delineated from the top of T9 to the top of the gluteus maximus dorsally and the pubic symphysis ventrally, with the posteroanterior boundary defined by the iliac bone. GFAT was segmented continuously from the ASAT boundary to the knee joint line.

Segmentation used an nnU-Net with a learning rate of 3 × 10⁻⁴, 1000 epochs, a patch size of 192 × 192 × 64 voxels, and on-the-fly affine, elastic, and gamma-intensity augmentations. A human-in-the-loop training strategy was employed. Initially, 20 manually segmented MRIs per cohort (40 total) were selected to reflect variation in body habitus and image quality and were used to train a preliminary model. Subsequent cases were selected iteratively to extend coverage of body habitus and image quality; their pre-segmentations were manually corrected and used for retraining. Because the four UK Biobank Dixon contrasts from the same acquisition were inherently aligned, each in-phase annotation was transferred directly to the opposed-phase, fat-only, and water-only reconstructions without image-registration software. Final development used 250 curated segmentation files: 50 NAKO T2-HASTE files and 200 UKB Dixon files from 50 UKB participants (four contrasts each). Five-fold cross-validation was participant-stratified. Manual segmentation and correction were performed in MONAI Label v0.7.0 by one first-year radiology resident (C.J.M.); these constituted the primary cross-validation references. Separately, two board-certified radiologists independently segmented a 50-scan subset drawn from UKB Dixon and NAKO T2-HASTE development-sequence images, blinded to the model output and to each other. Reader–reader and algorithm–reader Dice were summarized by compartment, and volume agreement was assessed by Bland–Altman analysis. Only aggregate statistics and de-identified plots are reported. The annotated NAKO development and reader-study images were T2-HASTE, whereas the population-scale NAKO compartment volumes were segmented from stitched in-phase GRE images. Thus, these agreement estimates do not directly validate inference on the NAKO GRE population input.

Liver fat fraction (LFF) was calculated from fat-only and water-only images^20,21^. Inference was performed on a single NVIDIA A100 GPU (approximately 60 seconds per scan). Automated checks flagged scans with implausible compartment volumes or truncated anatomy; flagged scans and a random sample passing automated checks were visually reviewed for anatomical plausibility. Quality-control findings are summarized under Statistics and reproducibility, with the targeted failure analysis in the Supplementary Information. Public inference for the 10-class body-composition task uses mrsegmentator --input <input > --outdir <output > --body_comp; the separate standard 40-class anatomical task used to obtain the liver mask is run with the same command without --body_comp. The public implementation used with the archived body-composition weights is pinned to MRSegmentator commit 8fd72378e7681cd0416318639102ec071c00d1af (https://github.com/hhaentze/MRSegmentator/tree/8fd72378e7681cd0416318639102ec071c00d1af). The standard nnU-Net v2 training entry point was nnUNetv2_train with the 3d_fullres configuration for folds 0–4; dataset.json, plans.json, fold-specific final checkpoints, and validation summaries are contained in the Zenodo weight archive. Cohort-preparation, harmonization, downstream analysis and figure-generation code and the trained weights are archived at https://doi.org/10.5281/zenodo.21211879.

### Classification of health behaviors and clinical conditions

In NAKO, handgrip strength was measured as the maximum isometric value from at least two attempts using a JamarPlus+ dynamometer, while physical activity was quantified by self-reported weekly metabolic equivalent of task (MET) minutes and categorized according to World Health Organization guidelines into four levels, ranging from insufficient ( < 600 MET min/week) through sufficient (600–999), moderate (1000–2999), to high (3000 + ). For UKB participants, data on smoking and alcohol intake frequency were available.

Clinical-condition classification was based on self-report from clinical interviews and included cardiometabolic, oncologic, and musculoskeletal diseases. Coronary artery disease (CAD) in NAKO was defined as a history of myocardial infarction, angina, or coronary stenosis; UKB used self-reported history of heart attack or angina. Type 2 diabetes was identified by self-report or, in NAKO, HbA1c ≥ 6.5% within three months before imaging. In both cohorts, hyperlipidemia was defined by self-reported high cholesterol or by any of the following laboratory thresholds: total cholesterol ≥ 240 mg/dl (6.21 mmol/l), LDL cholesterol ≥160 mg/dl (4.14 mmol/l), or triglycerides ≥ 200 mg/dl (2.26 mmol/l). No lipid-lowering-medication restriction was applied in the archived analysis code. Hypertension, osteoporosis, back pain, and gout were classified from self-reported medical history. Cancer was available only in UKB and was defined from self-reported cancer diagnoses (UKB field 20001); linked ICD cancer records were not used in the archived analysis.

### Data processing and statistical analysis

Volumetric features were harmonized with ComBat^22,23^ to minimize acquisition-related cohort shifts while preserving age-, sex-, height-, and BMI-related biological variation (Suppl. Fig. S2). Participants with missing covariate or outcome data were excluded from respective models. No imputation was performed.

Linear mixed-effects models examined associations between health behaviours and MRI-derived body-composition parameters, with fixed effects for standardized age, height, BMI, sex (in combined analyses), handgrip strength and physical activity (NAKO), or smoking and alcohol (UKB). Imaging centre was modelled as a random effect. Two-sided Wald *P* values for these exploratory health-behaviour models were adjusted using the Benjamini–Hochberg procedure (*α* = 0.05).

To investigate body composition independent of age, height, BMI, and sex, adjusted body composition parameters were derived from linear regression residuals. Associations between body composition and clinical conditions were estimated using generalized linear mixed-effects models, including the adjusted body composition parameters, age, BMI, and sex as fixed effects, with imaging center as a random effect. Odds ratios and predicted prevalence were calculated per one standard deviation increase in adjusted compartment volume. *P*-values for fixed effects were derived using Wald z-statistics. Because BMI and height were included as covariates, effect estimates for body composition compartments reflect differences in regional fat and muscle distribution for individuals with similar body size rather than effects of absolute changes in a single compartment^24,25^.

MRI-derived waist and hip circumferences were extracted from merged subcutaneous and gluteofemoral adipose-tissue contours. The waist plane was defined at L3 and the hip plane at the midpoint between the femoral heads and iliac bones; morphological closing and a convex hull produced continuous contours. The resulting waist-to-hip ratio (WHR) was validated against manual NAKO measurements (*n* = 3726 available; *r* = 0.94 for both waist and hip; Suppl.y Fig. S8). To evaluate discriminative value beyond traditional anthropometric measures, consecutive logistic mixed-effects models were constructed. Model performance was compared using area under the receiver operating characteristic curve (AUC) and change in AUC (ΔAUC; DeLong test), continuous net reclassification improvement (cNRI; Wald test), and likelihood-ratio tests (LRT). *P*-values for ΔAUC, cNRI, and LRT were adjusted using the Benjamini–Hochberg procedure at α = 0.05. Wald *P*-values for the primary compartment–condition association coefficients are reported without multiplicity adjustment.

### Statistics and reproducibility

All statistical analyses were performed in R version 4.4.3 using lme4^26^ for mixed-effects models, ggcorrplot for correlation matrices, forestplot and pheatmap for visualization of odds ratios, ggeffects^27^ for marginal predictions, and ggbump, fmsb, pROC, nricens, rmda, and DescTools for additional analyses. Cohort-characteristic plots were created using Python seaborn. A total of 250 curated segmentations were used for model training and five-fold cross-validation. No external test set was used. Systematic spot checks assessed anatomical plausibility and outlier volumes. A total of 76 scans ( < 0.2%) were flagged during segmentation quality control for anomalies, including truncated anatomy or implausible compartment boundaries. A targeted robustness analysis based on volumetric extremes and model-discordance screening is reported in the Supplementary Information. Sample sizes for each analysis are reported in the corresponding tables and figures.

## Results

### Segmentation performance and application scope

**Fig. 1:**
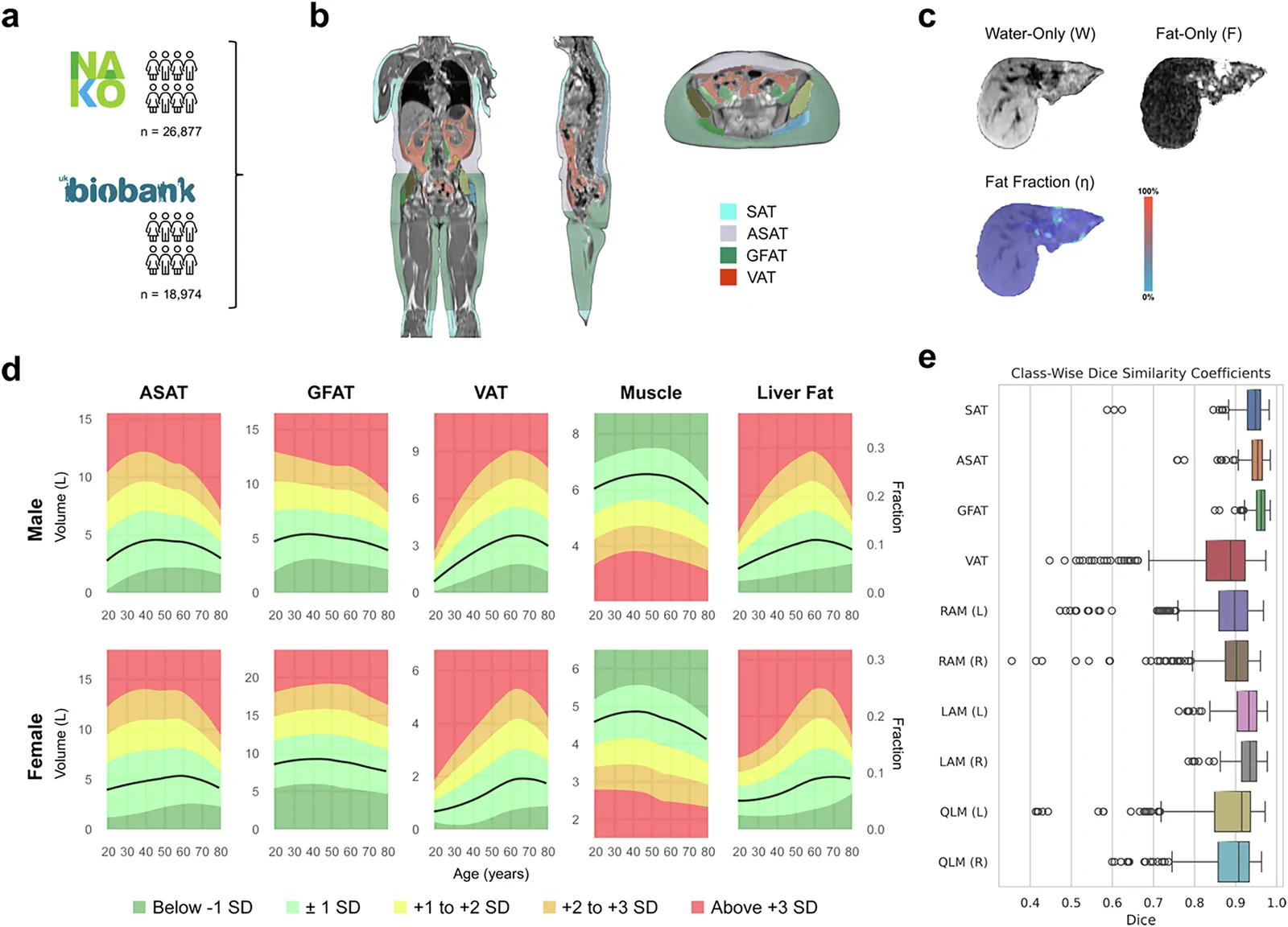
Deep learning-based segmentation of body composition from MRI scans. **a** A total of 26,877 NAKO and 18,974 UKB participants were included based on available imaging, lifestyle variables, and outcome data. **b** Representative segmentation results illustrate subcutaneous adipose tissue (SAT), abdominal subcutaneous adipose tissue (ASAT), gluteofemoral adipose tissue (GFAT), visceral adipose tissue (VAT), and trunk musculature, including rectus abdominis (RAM), lateral abdominal (LAM), and quadratus lumborum (QLM) muscles. **c** Liver images are shown with water-only, fat-only, and fat-fraction maps; fat fraction was calculated as η = F/(W + F). **d** Age-related patterns in body-composition parameters (BCPs) are shown as LOESS curves with pooled sex-specific means (black lines) and ± 1 standard deviation (shaded areas). **e** Dice similarity coefficients (DSC) for each segmented tissue class were evaluated by five-fold participant-stratified cross-validation on 250 curated segmentation files (50 NAKO T2-HASTE and 200 UKB Dixon files from 50 participants). Box plots display the median and interquartile range; whiskers extend to 1.5 times the IQR and points denote outliers. This panel is descriptive and no statistical test was applied. GFAT was not annotated in NAKO T2-HASTE. The NAKO T2-HASTE cross-validation estimates do not directly validate the stitched NAKO GRE input used for population phenotyping.

Within the annotated development data, five-fold participant-stratified internal cross-validation against the curated human-in-the-loop references yielded a mean Dice coefficient of 0.91 (range 0.85–0.96; Fig. 1e, Table 1). Mean Dice was 0.93 for NAKO T2-HASTE and 0.89–0.92 across the four UKB Dixon contrasts. In the separate 50-scan reader study drawn from UKB Dixon and NAKO T2-HASTE images, overall reader–reader Dice was 0.937 (95% CI 0.933–0.940) and overall algorithm–reader Dice was 0.908 (95% CI 0.905–0.912); compartment-specific Dice and Bland–Altman statistics are reported in Suppl. Table 12 and Fig. 12. The single model was subsequently used to segment population-scale compartment volumes from stitched in-phase GRE images in both cohorts; LFF was calculated from fat-only and water-only images. No external test set or annotated NAKO GRE reference set was evaluated. The cross-validation and reader-study results therefore quantify agreement on the annotated development-sequence domains rather than direct validation of the NAKO GRE population input.

**Table 1:** Dice Similarity Coefficients (DSC) for all classes

	SAT	ASAT	GFAT	VAT	RAM (L)	RAM (R)	LAM (L)	LAM (R)	QLM (L)	QLM (R)	Average
**F**	0.93 ± 0.05	0.95 ± 0.03	0.96 ± 0.02	0.85 ± 0.11	0.86 ± 0.10	0.88 ± 0.09	0.92 ± 0.04	0.93 ± 0.03	0.86 ± 0.10	0.88 ± 0.07	**0.90** ± **0.07**
**T2**	0.96 ± 0.02	0.96 ± 0.03	-	0.90 ± 0.05	0.91 ± 0.04	0.91 ± 0.05	0.95 ± 0.02	0.95 ± 0.03	0.92 ± 0.09	0.92 ± 0.06	**0.93** ± **0.04**
**W**	0.92 ± 0.05	0.94 ± 0.03	0.95 ± 0.02	0.81 ± 0.11	0.85 ± 0.10	0.86 ± 0.10	0.91 ± 0.04	0.92 ± 0.03	0.85 ± 0.11	0.87 ± 0.08	**0.89** ± **0.07**
**in**	0.95 ± 0.02	0.95 ± 0.02	0.97 ± 0.01	0.89 ± 0.06	0.89 ± 0.09	0.90 ± 0.08	0.93 ± 0.03	0.94 ± 0.03	0.87 ± 0.10	0.89 ± 0.07	**0.92** ± **0.05**
**opp**	0.93 ± 0.05	0.94 ± 0.03	0.96 ± 0.02	0.81 ± 0.13	0.84 ± 0.10	0.86 ± 0.10	0.91 ± 0.04	0.91 ± 0.03	0.85 ± 0.10	0.87 ± 0.08	**0.89** ± **0.07**
**Average**	**0.94** ± **0.04**	**0.95** ± **0.03**	**0.96** ± **0.02**	**0.85** ± **0.10**	**0.87** ± **0.09**	**0.88** ± **0.09**	**0.92** ± **0.04**	**0.93** ± **0.03**	**0.87** ± **0.10**	**0.88** ± **0.07**	**0.91** ± **0.06**

We used five-fold participant-stratified cross-validation on UKB Dixon in-phase, opposed-phase, water-only and fat-only (in, opp, W, F) and NAKO T2-HASTE data (T2). Classes are subcutaneous adipose tissue (SAT), abdominal subcutaneous adipose tissue (ASAT), gluteofemoral adipose tissue (GFAT), visceral adipose tissue (VAT), left and right rectus abdominis muscle (RAM), left and right lateral abdominal muscles (LAM), and left and right quadratus lumborum muscle (QLM). GFAT was not annotated in the original NAKO T2-HASTE development set; its cross-validation values are therefore based on UKB data only. The table shows mean DSC ± standard deviation.

Bold values are the average values.

### Cohort characteristics

A total of 45,851 adults were analyzed, comprising 26,877 (55.7% male) NAKO and 18,974 (47.9% male) UKB participants. Detailed cohort characteristics and disease prevalence are provided in Table 2. Median age was higher in UKB (63 years, IQR 56 to 68) than NAKO (48 years, IQR 40–58). BMI distributions were similar between cohorts (men 26.3 kg/m² in NAKO and 26.0 kg/m² in UKB, women 24.8 and 24.7 kg/m² respectively). Prevalence of CAD was 5.5% in UKB men and 1.2% in NAKO men. Type 2 diabetes prevalence was higher in UKB men (7.0% vs. 5.2%) but slightly lower in women (3.5% vs. 4.4%). Hyperlipidemia was more frequent in UKB (52.3% vs. 46.3% in men, 45.3% vs. 37.0% in women). Heart failure was more often reported in NAKO (1.3%) than UKB (0.1%), likely reflecting differences in questionnaire structure and diagnostic coding.

**Table 2:** Characteristics of NAKO and UKB participants

Dataset	NAKO		UKB	
Sex	Male (*n* = 14,969)	Female (*n* = 11,908)	Male (*n* = 9094)	Female (*n* = 9880)
Age (years)	48 [40,57]	49 [41,58]	64 [58,69]	62 [56,68]
Ethnicity				
White	14,148 (94.52%)	11,050 (92.79%)	8,779 (96.54%)	9,601 (97.18%)
Not specified	663 (4.43%)	730 (6.13%)		
Other*	158 (1.06%)	128 (1.07%)	315 (3.46%)	279 (2.82%)
Weight (kg)	85 [76,94]	68 [61,78]	82 [75,90]	66 [60,75]
Height (cm)	179 [174, 184]	166 [161, 170]	177 [173, 182]	163 [159, 168]
Body mass index (kg/m^2^)	26.3 [24.1, 29.1]	24.8 [22.1, 28.6]	26 [23.9, 28.5]	24.7 [22.4, 27.9]
Hand grip strength (kg)	48.7 [43.2, 54.7]	30.4 [26.7, 34.2]		
Physical activity (MET min. / week)				
Insufficient ( < 600)	2047 (13.67%)	1751 (14.7%)		
Sufficient (600–999)	931 (6.22%)	797 (6.69%)		
Moderate (1000–2999)	3770 (25.19%)	2954 (24.81%)		
High ( ≥ 3000)	8221 (54.92%)	6406 (53.8%)		
Alcohol				
Never			489 (5.38%)	720 (7.29%)
Special occasions only			589 (6.48%)	1359 (13.76%)
One to three times a month			864 (9.5%)	1361 (13.78%)
Once or twice a week			2373 (26.09%)	2736 (27.69%)
Three or four times a week			2903 (31.92%)	2413 (24.42%)
Daily or almost daily			1876 (20.63%)	1291 (13.07%)
Smoking				
Never			5265 (57.9%)	6532 (66.11%)
Previous			3414 (37.54%)	3021 (30.58%)
Current			415 (4.56%)	327 (3.31%)
**Body composition metrics**				
Total subcutaneous adipose tissue (Total SAT; L)	12.67 [9.58, 16.43]	17.79 [13.42, 23.49]	11.31 [8.65, 14.64]	17.79 [13.42, 23.49]
Abdominal subcutaneous adipose tissue (L)	3.98 [2.78, 5.55]	4.50 [2.99, 6.63]	3.47 [2.54, 4.77]	4.44 [3.11, 6.26]
Gluteofemoral adipose tissue (L)	4.82 [3.69, 6.26]	8.69 [6.81, 11.12]	4.16 [3.13, 5.55]	7.75 [5.93, 10.06]
Visceral adipose tissue (L)	2.69 [1.43, 4.14]	1.06 [0.59, 2.00]	3.10 [2.03, 4.38]	1.47 [0.98, 2.28]
Total trunk muscle volume (L)	6.38 [5.82, 7.00]	4.74 [4.34, 5.21]	6.21 [5.57, 6.91]	4.40 [3.97, 4.87]
Liver fat fraction (%)	7.45 [5.95, 10.87]	5.75 [4.71, 7.64]	8.44 [7.29, 10.65]	7.44 [6.52, 8.89]
**Disease outcomes**				
**Cardiovascular**				
Hypertension	3846 (25.69%)	2517 (21.14%)	2618 (28.79%)	1888 (19.11%)
CAD	180 (1.20%)	70 (0.59%)	501 (5.51%)	166 (1.68%)
Heart failure	189 (1.26%)	158 (1.33%)	9 (0.1%)	9 (0.09%)
**Metabolic**				
Type 2 diabetes	773 (5.16%)	529 (4.44%)	639 (7.03%)	349 (3.53%)
Hyperlipidaemia	6937 (46.34%)	4408 (37.02%)	4752 (52.25%)	4473 (45.27%)
Gout	781 (5.22%)	199 (1.67%)	270 (2.97%)	18 (0.18%)
**Other**				
Cancer			972 (10.69%)	1175 (11.89%)
Back pain	2854 (19.07%)	2837 (23.82%)	192 (2.11%)	187 (1.89%)
Osteoporosis	161 (1.08%)	403 (3.38%)	26 (0.29%)	285 (2.88%)

Note: Percentages may not sum to 100% because of rounding. Total SAT denotes the participant-level total subcutaneous adipose-tissue measure reported in Table 3; because medians are reported separately for each compartment, the displayed Total SAT median is not expected to equal the sum of the ASAT and GFAT medians.

Note: Continuous variables are shown as median with interquartile range in square brackets. Number of participants corresponding to a specific group are shown with relative abundance in percent.

*Other ethnicities specified in Suppl. Tables S1 and S2

### Body composition distribution by sex, age, and BMI

**Fig. 2:**
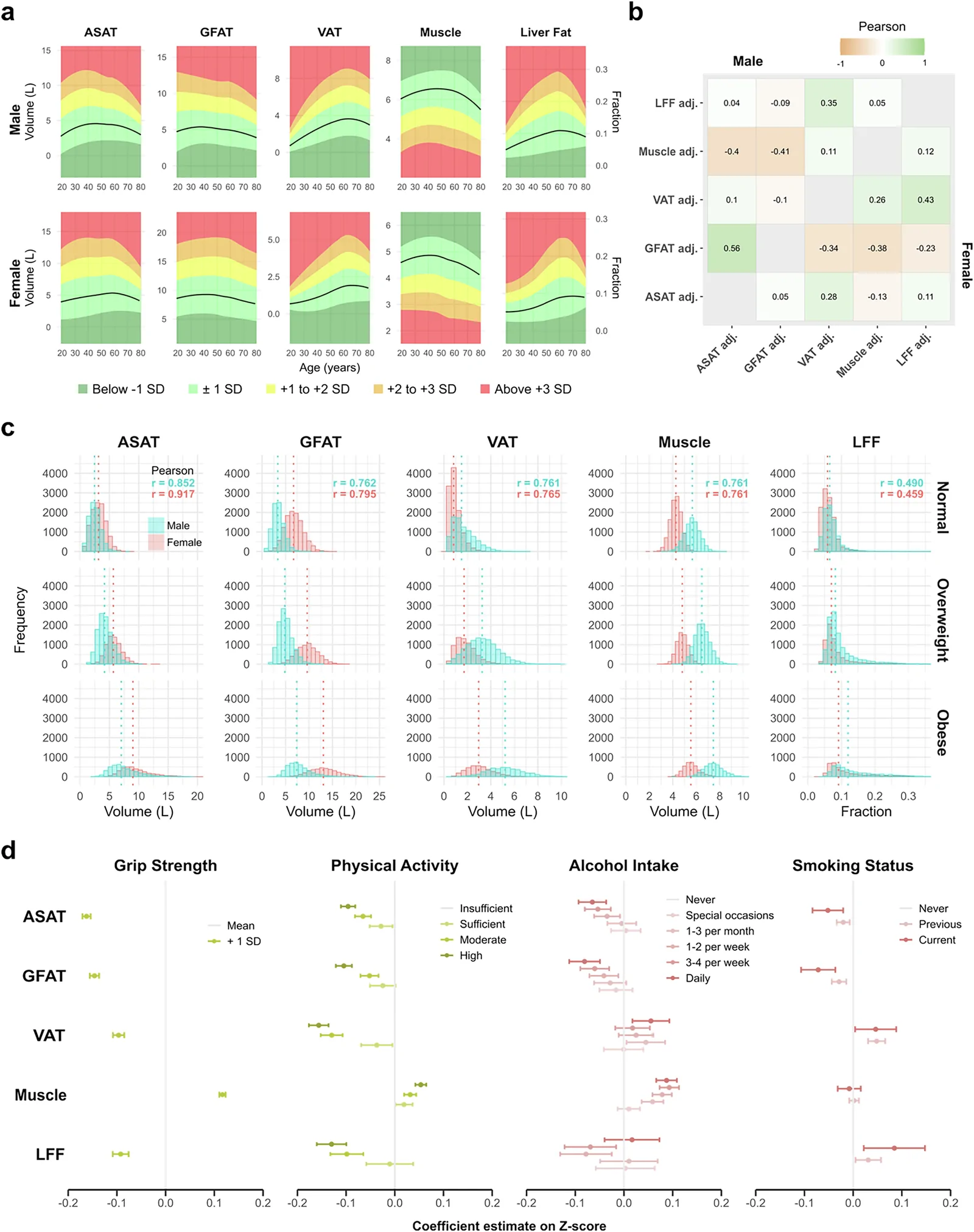
**Body-composition parameters and associations with age**. BMI, and health-behaviour indicators. **a** Age-related patterns in BCPs are shown as LOESS curves with pooled sex-specific means (black lines) and ± 1 standard deviation (shaded areas). **b** Correlations of adjusted BCPs are shown for men (upper triangle) and women (lower triangle); Pearson correlations were tested with two-sided t-tests. **c** Distributions of BCPs by BMI category are shown for men and women, with medians indicated by dashed lines. **d** Estimated associations between health-behaviour indicators and unadjusted BCP Z-scores were derived from linear mixed-effects models stratified by cohort and adjusted for age, height, BMI, sex where applicable, and available behavioural covariates, with imaging centre as a random intercept. n denotes participants (26,877 NAKO and 18,974 UKB). In (**d**), points show fixed-effect estimates, with error bars denoting 95% confidence intervals. *P*-values were derived from two-sided Wald tests and adjusted using the Benjamini–Hochberg procedure (*α* = 0.05); exact estimates and adjusted *P*-values are provided in Suppl. Tables S4–S5.

VAT increased markedly with age, with men showing higher overall levels than women. From ages 20–30 to ages 60–70, men exhibited a 236% increase while women showed a 179% increase (Fig. 2a, Table 3). Men also had higher trunk muscle volumes than women in both cohorts (*p* < 0.0001 for all comparisons). Women had higher GFAT than men and experienced smaller age-related declines (5% vs. 9%). These sex differences persisted across all BMI categories, including normal-weight individuals (Table 3, Suppl. Fig. S2), indicating systematic variation in compartmental fat and muscle distribution independent of BMI.

**Table 3:** Body composition distribution by sex, age, and BMI category

A. Body composition by sex and BMI category								
Sex	BMI Category	ASAT (L)		GFAT (L)	VAT (L)	Muscle (L)	LFF (%)	Total SAT (L)
Female	Normal *n* = 11,440	3.15 [2.27, 4.00]		6.74 [5.44, 8.11]	0.83 [0.58, 1.20]	4.27 [3.94, 4.59]	5.90 [4.72, 7.26]	13.49 [10.93, 15.94]
	Overweight *n* = 6569	5.68 [4.82, 6.61]		9.63 [8.04, 11.17]	1.72 [1.21, 2.35]	4.81 [4.48, 5.15]	7.05 [6.03, 8.88]	20.86 [18.56, 23.25]
	Obese *n* = 3779	9.00 [7.64, 10.86]		13.07 [11.01, 15.24]	2.96 [2.25, 3.74]	5.54 [5.14, 5.99]	9.17 [7.27, 14.31]	29.79 [26.51, 34.04]
Male	Normal *n* = 8706	2.46 [1.82, 3.11]		3.37 [2.63, 4.21]	1.50 [0.96, 2.28]	5.66 [5.23, 6.09]	6.56 [5.43, 7.99]	8.57 [6.82, 10.35]
	Overweight *n* = 10,964	4.16 [3.38, 5.07]		4.86 [3.96, 5.89]	3.26 [2.36, 4.19]	6.48 [6.06, 6.93]	8.23 [6.94, 10.92]	13.08 [11.13, 15.26]
	Obese *n* = 4393	7.02 [5.7, 8.84]		7.37 [6.02, 9.09]	5.21 [4.20, 6.31]	7.45 [6.94, 7.99]	12.03 [8.97, 18.28]	20.10 [17.05, 23.97]
**B. Body composition by sex and age group**								
Female	20–30 years *n* = 1389	3.43 [2.32, 5.35]		8.35 [6.75, 10.74]	0.58 [0.43, 0.90]	4.66 [4.29, 5.07]	4.55 [3.97, 5.35]	15.11 [11.67, 20.4]
	40–50 years *n* = 4442	4.31 [2.84, 6.46]		8.74 [6.82, 11.16]	0.99 [0.61, 1.75]	4.77 [4.34, 5.27]	5.72 [4.78, 7.19]	17.52 [13.15, 23.26]
	60–70 years *n* = 6632	4.71 [3.32, 6.53]		7.92 [6.05, 10.27]	1.62 [1.03, 2.53]	4.47 [4.05, 4.94]	7.52 [6.47, 9.37]	17.62 [13.54, 23.02]
Male	20–30 years *n* = 1880	2.87 [1.83, 4.53]		4.74 [3.36, 6.46]	1.03 [0.69, 1.82]	6.21 [5.69, 6.80]	5.36 [4.64, 6.52]	10.30 [7.11, 14.74]
	40–50 years *n* = 5253	4.03 [2.89, 5.68]		4.88 [3.75, 6.38]	2.71 [1.62, 3.99]	6.48 [5.92, 7.12]	7.38 [6.14, 10.41]	12.76 [9.78, 16.66]
	60–70 years *n* = 6702	3.77 [2.72, 5.09]		4.31 [3.28, 5.70]	3.46 [2.21, 4.78]	6.21 [5.61, 6.85]	8.69 [7.38, 11.82]	12.04 [9.22, 15.44]
**C. Age and sex effects**								
**Metric**	**Age Effect Female**		**Age Effect Male**		**Sex Effect Young Adult F/M**		**Sex Effect Older Adult F/M Ratio**	
ASAT	1.37		1.31		1.20		1.25	
GFAT	0.95		0.91		1.76		1.84	
VAT	2.79		3.36		0.56		0.47	
Muscle	0.96		1.00		0.75		0.72	
LFF	1.65		1.62		0.85		0.87	
Total SAT	1.17		1.17		1.47		1.46	

Table 3 integrates both BMI and age effects on body composition, providing a complete overview of the primary determinants of fat and muscle distribution. The table highlights how body fat compartments differ markedly between sexes, change systematically with aging, and vary across BMI categories. Panel A presents pooled median values and interquartile ranges (IQR) categorized by sex and BMI category based on harmonized data from the UK Biobank (UKB) and German National Cohort (NAKO). Panel B presents median and IQR by sex and age group. Panel C summarizes relative age-related changes and sex-specific ratios, showing percentage changes from early to late adulthood (20–30 years to 60–70 years) and female-to-male ratios in early and late adulthood.

Note: Values in Panel A and B represent median volumes and interquartile ranges (IQR). In Panel C, age effects represent relative changes from age 20–30 to age 60–70, and sex effects represent female-to-male ratios in the 20–30 and 60–70 age group respectively. *ASAT* abdominal subcutaneous adipose tissue, *GFAT* gluteofemoral adipose tissue, *VAT* visceral adipose tissue, *LFF* liver fat fraction, *Total SAT* total subcutaneous adipose tissue.

### Lifestyle-related differences in body composition

In NAKO, greater handgrip strength and higher physical activity were associated with lower VAT and higher muscle volume (Suppl. Table S4). Participants with moderate activity (1000 to 2999 MET min/week) had lower VAT Z-scores than insufficiently active participants ( − 0.13, 95% CI [ − 0.15, −0.11], *p* < 0.0001). The association exhibited diminishing returns at high activity levels ( − 0.156 for 3000 + MET min/week vs. −0.130 for 1000–2999). In UKB, current smoking was associated with lower ASAT ( − 0.052, 95% CI [ − 0.083, −0.020], *p* = 0.0019) and GFAT ( − 0.072, 95% CI [ − 0.107, −0.036], *p* = 0.0001) but higher VAT (0.046, 95% CI [0.004, 0.089], *p* = 0.043) and LFF (0.085, 95% CI [0.022, 0.148], p = 0.018), reflecting altered fat distribution among current smokers. These cross-sectional associations do not permit causal inference. Alcohol intake frequency showed inverse associations with subcutaneous fat compartments and positive associations with trunk muscle volume (Suppl. Table S5, Suppl. Fig. S3).

### Associations with cardiometabolic conditions

Residualized Z-scores of body composition parameters were derived by adjusting for age, sex, height, and BMI to reflect variation in fat and muscle distribution independent of these covariates (Suppl. Fig. S4). As BMI and height were included as covariates in all models, effect estimates reflect differences in regional distribution for individuals with similar body size rather than effects of absolute changes in a single compartment^24,25^. Increases in one compartment are therefore associated with redistribution from other compartments at constant BMI and height.

**Fig. 3:**
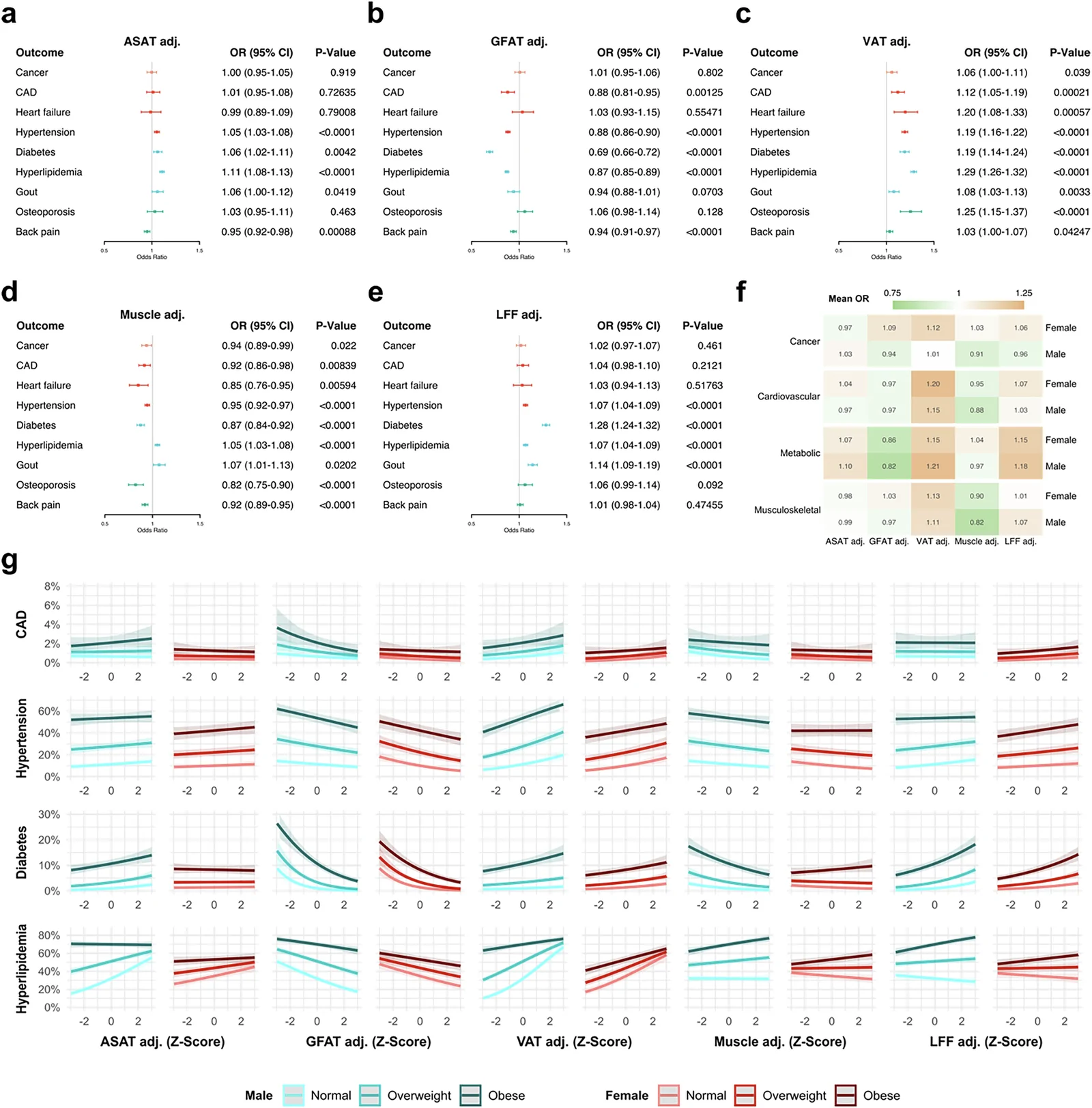
Associations between body composition parameters and clinical conditions. **a**–**e** Associations between adjusted body composition parameters (BCPs) and the prevalence of clinical conditions, presented as odds ratios (OR) derived from generalized mixed-effects logistic regression models. *P*-values for fixed effects were derived using Wald z-statistics. Models included adjusted BCPs, age, BMI, and sex, with imaging centre included as a random effect. Cancer data were available only in UKB participants. Colours indicate clinical condition categories. **f** Heatmap of average odds ratios for each clinical condition group, based on sex-stratified models (detailed results in Suppl. Fig. S5). **g** Estimated cross-sectional prevalence of selected conditions across adjusted BCP Z-scores, shown for BMI reference values of 20 kg/m² (normal), 27.5 kg/m² (overweight), and 35 kg/m² (obese). Estimates are based on marginalized means from sex-stratified generalized mixed-effects logistic regression models including interaction terms between BMI and each BCP, with imaging centre as a random effect. These models describe prevalence variation and should not be interpreted as predictive of future risk. Odds ratios in (**a**–**e**) are shown with 95% confidence intervals (error bars) from generalized linear mixed-effects models using two-sided Wald z-tests; n is the number of participants, with *n* = 45,851 (26,877 NAKO and 18,974 UK Biobank) for all conditions except cancer (UK Biobank only, *n* = 18,974). *P*-values are not adjusted for multiple comparisons (see Methods); exact values are in the Suppl. Information. In (**g**), lines show marginal predicted prevalence and shaded bands the 95% confidence interval.

In the pooled analysis, VAT showed the strongest positive associations with cardiometabolic conditions, with OR of 1.12 (95% CI 1.05–1.19) for CAD, 1.20 (95% CI 1.08–1.33) for heart failure, 1.19 (95% CI 1.16–1.22) for hypertension, 1.19 (95% CI 1.14–1.24) for type 2 diabetes, and 1.29 (95% CI 1.26–1.32) for hyperlipidemia. In the opposite direction, higher GFAT was associated with lower odds of type 2 diabetes (OR 0.69, 95% CI 0.66–0.72), CAD (OR 0.88, 95% CI 0.81–0.95), hypertension (OR 0.88, 95% CI 0.86–0.90), and hyperlipidemia (OR 0.87, 95% CI 0.85–0.89). ASAT was associated with higher odds of hypertension (OR 1.05, 95% CI 1.03–1.08), type 2 diabetes (OR 1.06, 95% CI 1.02–1.11), and hyperlipidemia (OR 1.11, 95% CI 1.08–1.13). Muscle showed inverse associations with type 2 diabetes (OR 0.87, 95% CI 0.84–0.92), heart failure (OR 0.85, 95% CI 0.76–0.96), and hypertension (OR 0.95, 95% CI 0.92–0.97). LFF was positively associated with hypertension, type 2 diabetes, and hyperlipidemia (Fig. 3a).

These compartment–disease patterns were descriptively internally consistent across cohorts, but no cohort-specific equivalence or heterogeneity test was performed. Conditions characterized by metabolic dysfunction showed a shared pooled phenotype of elevated VAT and LFF alongside reduced GFAT and muscle volume (Suppl. Fig. S6). Interaction models showed that adjusted body composition contributed to cross-sectional outcome discrimination across BMI categories (Fig. 3g, Suppl. Fig. S7).

Non-cardiometabolic associations followed similar patterns. Gout was associated with higher ASAT, VAT, muscle, and LFF and with lower GFAT. Osteoporosis showed opposing associations with VAT (OR 1.25, 95% CI 1.15–1.37) and muscle (OR 0.82, 95% CI 0.75–0.90). Back pain was modestly less common with higher GFAT (OR 0.94, 95% CI 0.91–0.97) and muscle (OR 0.92, 95% CI 0.89–0.95).

### Disease-specific body composition phenotypes

**Fig. 4:**
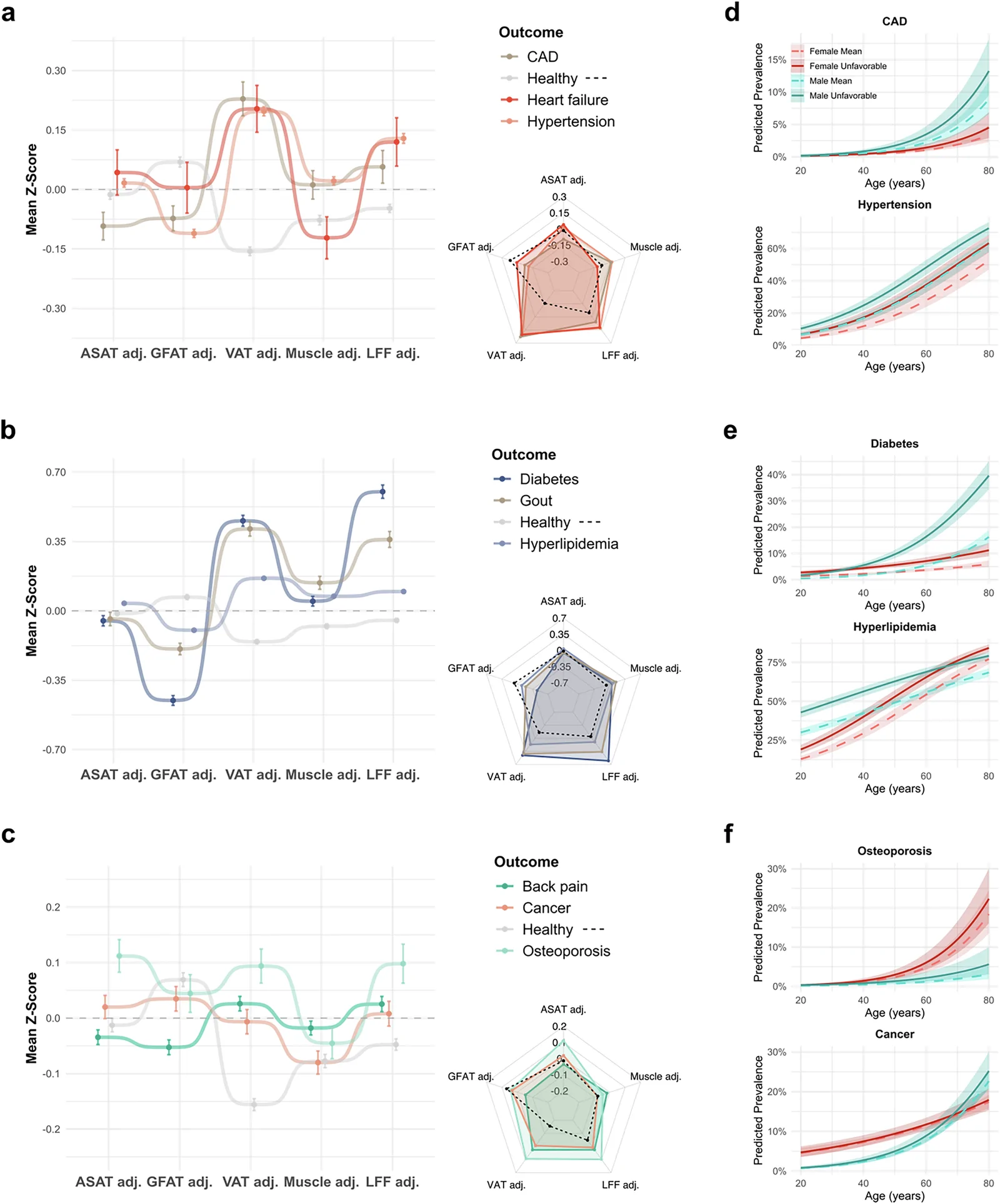
Body-composition phenotypes and their associations with prevalence of cardiometabolic, cancer, and related clinical conditions. **a**–**c** Mean Z-scores of adjusted body-composition parameters (BCPs) for participants with different clinical conditions are displayed using dot-whisker and spider plots. Values are means ± standard error. “Healthy” denotes UKB participants without any of the nine assessed conditions (*n* = 6393); because cancer status was unavailable in NAKO, this Healthy reference is UKB-only, whereas non-cancer condition groups otherwise pool both cohorts. **d**–**f** Estimated cross-sectional prevalence across age is shown for two standardized body-composition phenotypes: “unfavourable” (+1 SD in ASAT, VAT, and LFF; − 1 SD in GFAT and trunk muscle) and “mean” (Z = 0 for all BCPs). Estimates are marginalized predictions from sex-stratified generalized linear mixed-effects models adjusted for age, BMI, and adjusted BCPs, with imaging centre as a random effect. These models describe prevalence, not risk or prognosis. Cancer data were available only in UKB. n denotes participants; group sizes are given in Table 4. In (**a**–**c**), error bars show the standard error of the mean; in (**d**–**f**), shaded bands show 95% confidence intervals.

Distinct disease-specific body-composition phenotypes were observed when mean Z-scores of adjusted parameters were compared across conditions (Table 4, Fig. 4a–c). Individuals with type 2 diabetes had elevated VAT ( + 0.45 ± 1.31), reduced GFAT ( − 0.45 ± 1.18), and elevated LFF ( + 0.60 ± 1.61). Hypertensive individuals had higher VAT ( + 0.20 ± 1.19) and slightly lower GFAT ( − 0.11 ± 1.07) than the Healthy reference (VAT − 0.16 ± 0.86, GFAT + 0.07 ± 1.00). Because cancer was unavailable in NAKO and the Healthy indicator required absence of all nine assessed conditions, the Healthy reference used for Table 4 and Fig. 4a–c comprised UKB participants only (*n* = 6393); the non-cancer condition groups otherwise used the pooled cohorts. Cancer cases showed more subtle alterations that varied by sex (Suppl. Fig. S6).

**Table 4:** Body composition Z-scores by clinical condition

Clinical Condition	*n*	Mean Z-Scores				
		ASAT adj.	GFAT adj.	VAT adj.	Muscle adj.	LFF adj.
**Cardiovascular**						
CAD	917	−0.09 ± 1.06 (0.03)	−0.07 ± 0.96 (0.03)	0.23 ± 1.29 (0.04)	0.01 ± 1.09 (0.04)	0.06 ± 1.25 (0.04)
Heart failure	365	0.04 ± 1.09 (0.06)	0.00 ± 1.22 (0.06)	0.20 ± 1.13 (0.06)	−0.12 ± 1.01 (0.05)	0.12 ± 1.16 (0.06)
Hypertension	10,869	0.02 ± 1.11 (0.01)	−0.11 ± 1.07 (0.01)	0.20 ± 1.19 (0.01)	0.02 ± 1.09 (0.01)	0.13 ± 1.29 (0.01)
**Metabolic**						
Diabetes	2290	−0.05 ± 1.25 (0.03)	−0.45 ± 1.18 (0.02)	0.45 ± 1.31 (0.03)	0.05 ± 1.17 (0.02)	0.60 ± 1.61 (0.03)
Hyperlipidaemia	20,570	0.04 ± 1.02 (0.01)	−0.10 ± 1.00 (0.01)	0.16 ± 1.05 (0.01)	0.07 ± 1.02 (0.01)	0.10 ± 1.14 (0.01)
Gout	1268	−0.04 ± 1.21 (0.03)	−0.19 ± 1.04 (0.03)	0.41 ± 1.30 (0.04)	0.14 ± 1.17 (0.03)	0.36 ± 1.44 (0.04)
**Other**						
Back pain	6,070	−0.03 ± 1.03 (0.01)	−0.05 ± 1.04 (0.01)	0.03 ± 1.02 (0.01)	−0.02 ± 0.98 (0.01)	0.03 ± 1.07 (0.01)
Cancer	2147	0.02 ± 0.97 (0.02)	0.03 ± 1.02 (0.02)	−0.01 ± 1.01 (0.02)	−0.08 ± 0.96 (0.02)	0.01 ± 1.03 (0.02)
Osteoporosis	875	0.11 ± 0.87 (0.03)	0.04 ± 1.01 (0.03)	0.09 ± 0.91 (0.03)	−0.05 ± 0.82 (0.03)	0.10 ± 1.04 (0.04)
**Reference**						
Healthy (UKB only)	6393	−0.01 ± 0.96 (0.01)	0.07 ± 1.00 (0.01)	−0.16 ± 0.86 (0.01)	−0.08 ± 0.96 (0.01)	−0.05 ± 0.82 (0.01)

This table presents mean Z-scores of adjusted body composition parameters (abdominal subcutaneous adipose tissue [ASAT], gluteofemoral adipose tissue [GFAT], visceral adipose tissue [VAT], trunk muscle volume, and liver fat fraction [LFF]) across multiple clinical conditions, as determined in the provided dataset. Each row corresponds to a specific disease/condition category, listing the number of individuals (n), followed by the mean Z-score and standard deviation (with standard error in parentheses) for each parameter. Positive Z-scores indicate higher relative values compared to the average total population, whereas negative values indicate lower relative values.

Note: Values represent mean ± standard deviation with standard error in parentheses. Positive values indicate higher relative values compared to the total population. *ASAT* abdominal subcutaneous adipose tissue, *GFAT* gluteofemoral adipose tissue, *VAT* visceral adipose tissue, *LFF* liver fat fraction, *adj.* adjusted.

Based on these patterns, an “unfavourable” body-composition profile was defined as +1 SD in ASAT, VAT, and LFF combined with −1 SD in GFAT and muscle. Predicted prevalence across age for this profile and for a mean profile (Z = 0 for all parameters) is shown in Fig. 4d–f; age- and sex-stratified prevalence estimates for all clinical conditions are summarized in Table 5. Women with the unfavourable profile reached hyperlipidemia prevalence nearly a decade earlier than men with average body composition, illustrating the added value of regional phenotyping for understanding heterogeneity within BMI categories.

**Table 5:** Predicted prevalence of cardiometabolic, cancer, and related clinical conditions by sex and age

Clinical Condition	Sex	Age Group	Predicted Prevalence (%)	Sex Ratio (F/M)
**Cardiovascular Conditions**				
CAD	Female	20–40 years	0.23 (0.06–0.65)	0.61
		41–60 years	0.75 (0.25–1.99)	0.47
		61–80 years	2.40 (0.86–6.90)	0.38
	Male	20–40 years	0.37 (0.09–1.19)	1.00
		41-60 years	1.59 (0.43–4.73)	1.00
		61-80 years	6.33 (1.87–18.17)	1.00
Hypertension	Female	20-40 years	9.55 (3.62–20.77)	0.68
		41–60 years	23.29 (10.34–42.89)	0.74
		61–80 years	45.75 (24.85–68.59)	0.82
	Male	20–40 years	14.03 (5.71–28.28)	1.00
		41–60 years	31.58 (15.52–52.83)	1.00
		61–80 years	55.83 (34.23–76.33)	1.00
**Metabolic Conditions**				
Diabetes	Female	20–40 years	2.66 (1.10–5.40)	1.24
		41–60 years	4.35 (1.97–8.58)	0.63
		61–80 years	6.96 (3.20–13.99)	0.37
	Male	20–40 years	2.15 (0.40–6.65)	1.00
		41–60 years	6.87 (1.53–19.04)	1.00
		61–80 years	18.92 (5.08–45.10)	1.00
Hyperlipidaemia	Female	20–40 years	24.65 (11.13–44.21)	0.58
		41–60 years	47.90 (27.47–69.26)	0.85
		61–80 years	71.60 (51.72–86.63)	1.04
	Male	20–40 years	42.85 (27.61–59.48)	1.00
		41–60 years	56.32 (40.53–71.70)	1.00
		61–80 years	68.65 (53.88–81.54)	1.00
**Other Conditions**				
Osteoporosis	Female	20–40 years	0.67 (0.17–2.09)	1.68
		41-60 years	2.99 (0.86–8.53)	2.74
		61–80 years	11.90 (3.79–30.04)	4.13
	Male	20–40 years	0.40 (0.09–1.44)	1.00
		41–60 years	1.09 (0.29–3.72)	1.00
		61–80 years	2.88 (0.79–10.03)	1.00
Cancer	Female	20–40 years	5.90 (3.54–9.03)	3.95
		41–60 years	9.43 (6.37–13.43)	1.86
		61–80 years	14.58 (10.46–20.81)	0.95
	Male	20–40 years	1.49 (0.49–3.63)	1.00
		41–60 years	5.08 (2.06–10.81)	1.00
		61–80 years	15.42 (7.65–30.05)	1.00

This table presents the predicted prevalence of cardiometabolic, cancer, and related clinical conditions for three age groups (20–40, 41–60, 61–80 years) in females and males.

Note: Mean predicted prevalence was multiplied by 100 and is presented with the smallest lower confidence bound and largest upper confidence bound observed in each group. Sex Ratio (F/M) is the female mean divided by the male mean prevalence for the matching age range.

### Associations with cancer prevalence

In UKB, where cancer status was available, associations between body composition and cancer prevalence were weaker and less consistent than for cardiometabolic outcomes. Higher VAT was associated with higher cancer prevalence in women (OR 1.12, 95% CI 1.05–1.21, *p* = 0.0012) but not men. Muscle showed an inverse association with cancer prevalence overall (OR 0.94, 95% CI 0.89–0.99, *p* = 0.022), driven by men (OR 0.91, 95% CI 0.85–0.99, *p* = 0.026). Adjusted body composition differences between cancer cases and controls were modest (Suppl. Fig. S6).

### Incremental discrimination beyond anthropometric measures

**Fig. 5:**
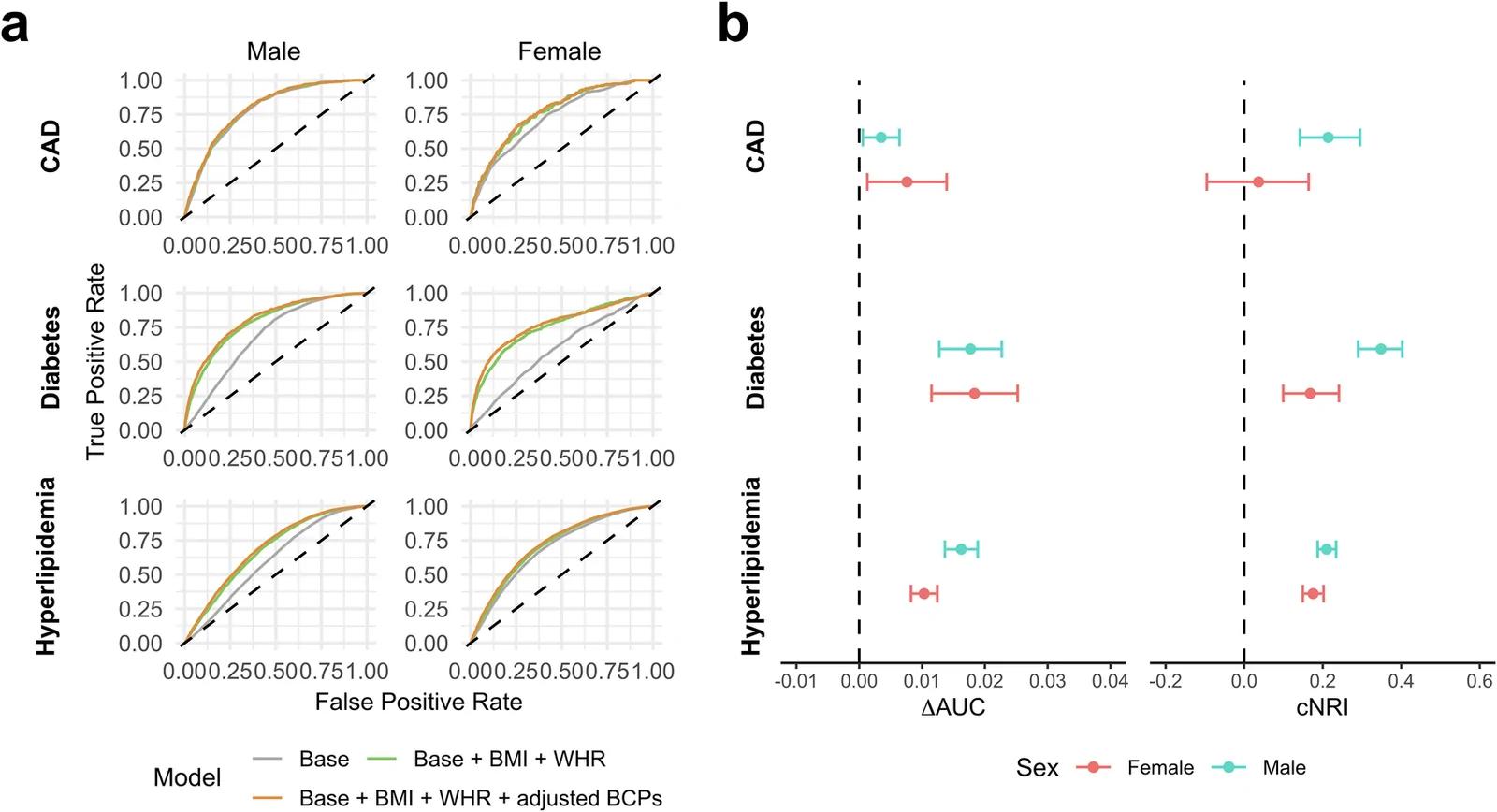
Discriminative value of MRI-derived body composition beyond traditional anthropometric measures. **a** Receiver operating characteristic (ROC) curves from generalized linear mixed-effects models of coronary artery disease (CAD), type 2 diabetes, and hyperlipidemia in male (*n* = 23,651) and female (*n* = 21,587) participants with MRI-derived waist-to-hip ratio (WHR). Shown are base models (age only), models adding anthropometric variables (BMI and WHR), and models further including adjusted body composition parameters (adjusted BCPs); ROC curves for the remaining outcomes are provided in Suppl. Fig. S9. **b** ΔAUC and continuous net reclassification improvement (cNRI) with 95% confidence intervals comparing models including age, BMI, and WHR versus models additionally including adjusted BCPs; higher cNRI indicates improved classification when adding BCPs. Calibration curves and decision-curve analyses are provided in Suppl. Figs. S10, S11, and numeric data in Suppl. Tables S7, S8. n denotes the number of participants; ΔAUC was compared with the two-sided DeLong test and cNRI with a two-sided Wald test, with *P*-values adjusted across outcomes by the Benjamini–Hochberg procedure (*α* = 0.05). The figure file is provided separately as Fig. 5.

Consecutive logistic regression models assessed whether MRI-derived compartments improved discrimination beyond age, BMI, and MRI-derived waist-to-hip ratio (WHR). Model statistics are available in Suppl. Tables S7, S8, with ROC curves in Fig. 5a (with additional outcomes in Suppl. Fig. S9), and calibration and decision-curve analyses in Suppl. Figs. S10, S11.

Adding MRI-derived body composition to age+BMI + WHR models improved discrimination for type 2 diabetes (ΔAUC men 0.018, women 0.018, both *p* < 0.001) and hyperlipidemia (ΔAUC men 0.016, women 0.010, both *p* < 0.001), with smaller gains for CAD (ΔAUC men 0.004, *p* = 0.026, and women 0.008, *p* = 0.027). Calibration slopes remained near one across models, and Brier scores decreased with the addition of MRI compartments. Likelihood ratio tests confirmed improved model fit for most cardiometabolic outcomes. Decision curve analysis showed net benefit from adding MRI compartments, particularly for diabetes (Suppl. Fig. S11).

These gains are modest in absolute terms, consistent with the study design. BMI and WHR already capture substantial variance in body size, while MRI compartments add information about fat and muscle distribution at constant body size. The incremental signal reflects distributional variation that anthropometric indices cannot resolve. For cancer, ΔAUC values were below 0.01, cNRI reached significance only in women (0.062, *p* = 0.075 in men, and 0.088, *p* = 0.008 in women), and likelihood ratio tests showed no improvement in model fit for men (*p* = 0.270, women *p* < 0.001).

## Discussion

This study applied a single open-source, multi-sequence deep-learning pipeline to large MRI datasets from two European cohorts and enabled pooled phenotyping of 45,851 participants. In the annotated development data, participant-stratified internal cross-validation against curated human-in-the-loop references yielded a mean Dice of 0.91 across UKB Dixon and NAKO T2-HASTE inputs (Table 1), with inference taking approximately one minute per scan. A separate 50-scan two-reader study on these development-sequence images yielded overall reader–reader and algorithm–reader Dice values of 0.937 and 0.908, respectively. Population-scale compartment volumes were segmented from stitched in-phase GRE images in both cohorts, while LFF was calculated from fat-only and water-only images. These results establish high agreement on the annotated development sequences; sequence-matched NAKO GRE and broader scanner validation are important next steps. The open-source availability of MRSegmentator^15^ enables independent validation, adaptation to other imaging protocols, and integration into research pipelines.

The pooled compartment–disease associations aligned with prior CT and MRI literature despite differences between the contributing cohorts. VAT showed the strongest positive associations with cardiometabolic conditions, while GFAT and trunk-muscle volume were inversely associated; GFAT showed the largest inverse association with type 2 diabetes (OR 0.69). Similar patterns have been reported in CT-based body-composition studies in more than 100,000 patients^7,8,10^ and in prior MRI work in UK Biobank^1,14^. This convergence supports the biological plausibility of the observed associations, but it does not demonstrate cohort equivalence, sequence-level generalization, or measurement validity on the unannotated NAKO GRE population input. Biologically, the patterns are compatible with the distinct metabolic roles of the depots: visceral and hepatic fat are lipolytically active and promote insulin resistance, low-grade inflammation, and ectopic lipid deposition, whereas gluteofemoral fat can act as a long-term lipid reservoir, and skeletal muscle supports insulin-mediated glucose disposal.

MRI-based body-composition analysis complements rather than competes with CT-based approaches. Three capabilities distinguish MRI in this context. First, GFAT can be quantified directly from whole-body MRI acquisitions, whereas axial CT protocols typically do not extend to the gluteofemoral region. GFAT showed among the strongest inverse associations in this study, suggesting that it carries clinically relevant distributional information that CT-based pipelines cannot access. Second, trunk-muscle volume is measured volumetrically from MRI rather than estimated through attenuation-based surrogates at a single vertebral level. Third, the absence of ionizing radiation makes MRI suitable for longitudinal monitoring and screening in healthy populations, a prerequisite for population-based cohort studies such as NAKO and UKB.

The identification of disease-specific body composition phenotypes adds translational value beyond individual compartment-disease associations. Type 2 diabetes was characterized by a consistent pattern of elevated VAT and LFF alongside reduced GFAT and muscle, while cancer showed more subtle and sex-specific alterations. The “unfavorable” profile analysis demonstrated that women with high VAT and low muscle mass reached hyperlipidemia prevalence nearly a decade earlier than men with average body composition, illustrating the clinical relevance of regional phenotyping for understanding heterogeneity within BMI categories.

This study extends prior MRI-based body-composition work in several ways. Agrawal et al. used 2D MRI projections to estimate fat-depot volumes and demonstrated BMI-adjusted associations with cardiometabolic disease^1,14^. Linge et al. applied a commercial platform (AMRA Researcher) to analyse fat distribution and incident outcomes^28^. More recently, whole-body MRI-derived body-composition reference curves have been reported in more than 66,000 individuals^29^. The present approach differs by performing full 3D volumetric segmentation rather than 2D projections, applying a pooled model across two cohorts with different imaging protocols, including trunk-muscle compartments that showed independent inverse associations with cardiometabolic conditions, and providing an open-source inference framework. The incremental-discrimination analysis further demonstrates that MRI-derived compartments provide measurable added value beyond conventional anthropometric measures for metabolic outcomes.

This study has limitations. The cross-sectional design precludes causal inference, and the absence of incident cardiovascular outcomes means that associations reflect prevalent disease rather than prospective risk. Reverse causation is possible, particularly for conditions such as cancer or heart failure, where disease may influence body composition. Longitudinal follow-up with adjudicated endpoints will be essential to establish predictive utility. Clinical conditions were classified by self-report, which may introduce misclassification, although laboratory confirmation for hyperlipidemia and diabetes in NAKO partially mitigated this concern. Both cohorts comprise predominantly middle-aged adults of European descent who volunteered for imaging, which may limit generalizability. Cancer endpoints were available only in UKB, and the observed associations were weaker and less consistent than for cardiometabolic outcomes. The modest absolute discrimination gains (ΔAUC 0.01–0.02) over models already containing BMI and WHR indicate that MRI compartments add distributional information rather than replace body-size measures. Segmentation accuracy was quantified primarily against curated development references comprising initially manual segmentations and subsequent manually corrected pre-segmentations; this human-in-the-loop design may bias cross-validation agreement upward. A separate two-reader study quantified reader–reader and algorithm–reader agreement on 50 UKB Dixon and NAKO T2-HASTE images, but it did not assess the NAKO GRE population input. Targeted review of volumetric extremes and model-discordant cases supported overall segmentation reliability while identifying specific failure modes: SAT underestimation when body girth exceeded the MRI field of view in nine participants with BMI > 40 kg/m², and occasional GFAT under- or over-segmentation in very lean NAKO participants. Dedicated stress testing for substantial motion artefact and severe muscle atrophy was not performed and remains warranted.

In conclusion, a single open-source pipeline enabled pooled MRI body-composition phenotyping in two large cohorts. Five-fold internal cross-validation against curated human-in-the-loop UKB Dixon and NAKO T2-HASTE references yielded a mean Dice of 0.91, and a separate two-reader study demonstrated high agreement on the same development-sequence domains. Disease-specific phenotypes provide translational context for the cross-sectional associations, and the incremental-discrimination analysis shows modest added value for selected metabolic outcomes. Together, these findings establish a practical framework for scalable MRI body-composition phenotyping. Future studies can integrate MRI-derived compartments with genetic, lifestyle, and biomarker data to model incident outcomes and support individualized risk assessment.

## Supplementary information


Supplementary Information
Description of Additional Supplementary files
Supplementary Data 1
Supplementary Data 2


## Data Availability

The individual-participant data underlying this study were obtained from the UK Biobank (https://www.ukbiobank.ac.uk) under application 34479 and from the German National Cohort (NAKO; https://nako.de) under data-access application NAKO-836; they comprise whole-body MRI together with questionnaire-, examination-, and laboratory-derived health data. These primary data cannot be shared publicly because of participant-privacy, data-protection, and cohort data-use restrictions; bona fide researchers may apply directly to the cohorts. During the study, data were held on secure, access-controlled servers at the Technical University of Munich. Processed, de-identified body-composition measurements are available from the corresponding author (L.C.A.) upon reasonable request and subject to cohort approval and data-use agreements; requests will receive a response within eight weeks. Non-identifying numerical Source Data corresponding to the quantitative panels in Figs. 1–5 are supplied in Suppl. Data 1, with figure captions and a panel-level data map. The workbook contains aggregate summaries, model estimates, prediction grids, and plot-resolution ROC coordinates; exact threshold-level Fig. 5a ROC coordinates are supplied in Suppl. Data 2. For Fig. 1e, exact sequence-by-class mean and standard-deviation values are supplied without case-level segmentation outputs. No raw images, participant identifiers, or individual-level cohort records are included. Cohort-preparation, harmonization, downstream analysis and figure-generation code and trained model weights are openly available at Zenodo (https://doi.org/10.5281/zenodo.21211879). The archived notebooks begin from approved cohort extracts and derived measurements; they do not redistribute raw images or individual-level records. The UK Biobank and NAKO study designs and imaging resources have been detailed in the respective cohort publications^16–18,30^.

## Code availability

MRSegmentator is openly available under an open-source licence, the version used in this work is available at https://github.com/hhaentze/MRSegmentator/tree/8fd72378e7681cd0416318639102ec071c00d1af. Standard nnU-Net v2 training used nnUNetv2_train with the 3d_fullres configuration for folds 0–4. Cohort-preparation, harmonization, downstream analysis and figure-generation code are archived at Zenodo (https://doi.org/10.5281/zenodo.21211879). Statistical analyses used R version 4.4.3 with the packages listed in the Methods.
