# Supplementary material for: Pooled two-cohort MRI body composition phenotyping with open-source deep learning
Source: Commun Med (Lond). 2026 Aug 29;6:467. doi: 10.1038/s43856-026-01888-w (PMC13526011; doi:10.1038/s43856-026-01888-w)
Supplement: Supplementary file 2 [file 43856_2026_1888_MOESM2_ESM.docx]

**Description of Additional Supplementary Files**

File name: Supplementary Data 1

Description: Non-identifying, panel-matched numerical values underlying the quantitative panels in Figures 1–5, including cohort counts, aggregate Dice summaries, age trends, correlations, distribution summaries, model estimates, association estimates, prevalence curves, phenotype summaries, ROC/AUC, ΔAUC, and cNRI results. Exact threshold-level Figure 5a ROC coordinates are provided in the companion CSV; no raw images or participant-level records are included.

File name: Supplementary Data 2

Description: Exact threshold-level false-positive-rate and true-positive-rate coordinates underlying Figure 5a for coronary artery disease, type 2 diabetes, and hyperlipidemia, stratified by sex and model. Contains aggregate curve data only and no participant-level records.
