# Supplementary material for: Pooled two-cohort MRI body composition phenotyping with open-source deep learning
Source: Commun Med (Lond). 2026 Aug 29;6:467. doi: 10.1038/s43856-026-01888-w (PMC13526011; doi:10.1038/s43856-026-01888-w)
Supplement: Supplementary file 3 [file 43856_2026_1888_MOESM1_ESM.docx]

Supplementary Material and Methods
MRI Cohorts and Image Acquisition 
Body-composition segmentation used the 10-class MRI body-composition task in MRSegmentator, an open-source nnU-Net-based pipeline. This task outputs subcutaneous and visceral fat, left and right rectus abdominis, oblique, and quadratus lumborum muscles, abdominal subcutaneous fat, and gluteofemoral fat; bilateral muscle classes were aggregated for analysis. The standard 40-class anatomical task separately supplied the liver mask for liver fat-fraction estimation. Thus, the body-composition outputs are an additional task rather than replacements for the standard anatomical classes. The body-composition task was trained only on UK Biobank and NAKO data; the anatomical task used for the liver mask had additional training data, as described in ref. 15 of the main manuscript. Initial training used 20 manually segmented scans per cohort; subsequent pre-segmentations were corrected in a human-in-the-loop workflow using MONAI Label v0.7.0 by one first-year radiology resident (C.J.M.). At participant level, 50 NAKO and 50 UK Biobank cases were used. Each UK Biobank in-phase annotation was transferred directly to the inherently aligned opposed-phase, fat-only, and water-only reconstructions, yielding 200 UK Biobank segmentation files; no image-registration software was used. Final development therefore used 250 curated files (50 NAKO T2-HASTE and 200 UKB Dixon reconstructions) with five-fold participant-stratified cross-validation. Separately, two board-certified radiologists independently segmented a 50-scan subset drawn from UKB Dixon and NAKO T2-HASTE development-sequence images for the reader–reader analysis. The model used a learning rate of 3 × 10⁻⁴, 1,000 epochs, 192 × 192 × 64-voxel patches, and on-the-fly affine, elastic, and gamma-intensity augmentation. For the population analysis, compartment volumes in both cohorts were segmented from stitched in-phase GRE images, while liver fat fraction was calculated from fat-only and water-only images. The annotated NAKO development and reader-study images were T2-HASTE; consequently, these agreement results do not directly validate the NAKO GRE population input.
Statistical analyses used R version 4.4.3. Linear mixed-effects models were implemented using lme4; correlation matrices used ggcorrplot; marginal predictions used ggeffects; forest plots and heatmaps used forestplot and pheatmap; and additional visualizations used ggbump, fmsb, pROC, nricens, rmda, DescTools, and Python seaborn. Unless otherwise stated, tests were two-sided. Wald P values for the primary compartment–condition association coefficients were not adjusted for multiple comparisons. Exploratory health-behaviour analyses in Supplementary Tables 4 and 5 and discrimination-model comparisons in Supplementary Tables 7 and 8 used Benjamini–Hochberg adjustment as stated in their notes. ComBat harmonization followed Gatidis et al. (1) and Johnson et al. (2).
MRSegmentator code for inference with the archived body-composition weights is pinned to commit 8fd72378e7681cd0416318639102ec071c00d1af (https://github.com/hhaentze/MRSegmentator/tree/8fd72378e7681cd0416318639102ec071c00d1af). The 10-class body-composition task uses --body_comp; the separate standard 40-class anatomical task used for the liver mask runs without this option. Standard nnU-Net v2 training used nnUNetv2_train with the 3d_fullres configuration for folds 0–4. Cohort-preparation, harmonization, downstream analysis and figure-generation code, dataset.json, plans.json, five final fold checkpoints, validation summaries, and trained weights are archived at https://doi.org/10.5281/zenodo.21211879. Participant-level UK Biobank and NAKO data remain subject to the cohorts’ access procedures and cannot be redistributed by the authors.

Segmentation failure analysis
To identify rare failure cases and assess robustness at the extremes, we implemented two complementary quality control procedures: volumetric outlier inspection and ensemble-based uncertainty estimation.
For volumetric inspection, we identified the highest and lowest volume segmentations for VAT, GFAT, SAT, combined musculature, and liver in both cohorts. Visual review confirmed accurate VAT, muscle, and liver segmentations across all extremes. SAT was also correctly segmented in low-BMI participants. However, SAT volume was underestimated in nine participants with extreme obesity (BMI > 40 kg/m²), where body girth exceeded the MRI field of view. GFAT segmentation was consistently accurate in UKB. In contrast, in NAKO, several low-volume scans, primarily in very lean participants, showed under- or over-segmentation near the thighs, likely due to insufficient contrast in the GFAT layer at low fat volumes.
To further probe segmentation reliability, we used a model-discordance heuristic based on Kofler et al.’s proposal to use disagreement within segmentation ensembles as an unsupervised quality estimate (4). We randomly selected 1,000 participants from each cohort to limit computational and environmental costs, then applied two nnU-Net sub-models trained on different folds. Dice similarity between model outputs was used to identify the lowest-agreement cases for manual review.
Most discrepancies involved subtle boundary shifts between GFAT, SAT, and ASAT, where anatomical transitions are gradual and poorly demarcated. These variations were deemed acceptable on visual inspection. No systematic errors were observed in the UKB cohort. In NAKO, consistent with volumetric findings, the only recurrent issue was under-segmentation of GFAT in individuals with very low thigh fat.
In summary, the targeted quality-control review supported overall segmentation reliability within the inspected extremes while identifying specific failure modes: SAT underestimation when body girth exceeded the field of view in nine participants with BMI > 40 kg/m², and occasional GFAT under- or over-segmentation in very lean NAKO participants. The review was not a prespecified stress test for substantial motion artefact or severe muscle atrophy, and the recurrent NAKO GFAT pattern should not be interpreted as randomly distributed across all inputs.

Waist and Hip Circumference Extraction
Joint hip and waist measurements were available for 3,726 participants in the NAKO data and for none of the UKB data. Research has shown that measuring waist circumference in CT volumes closely correlates with manually measured values (3), and this likely transfers to MRI as well. For the hip, we defined the axial measurement plane as the midpoint between the left and right femoral heads and the iliac bones. For the waist, the measurement plane was defined at the level of the third lumbar vertebra (L3), based on the WHO protocol to measure "at the approximate midpoint between the lower margin of the last palpable rib and the top of iliac crest" (https://www.who.int/publications/i/item/9789241501491) (Supplementary Figure 8). 
To simulate tape-based circumference measurements, we calculated perimeters on axial slices at these predefined locations using merged subcutaneous and gluteofemoral adipose tissue. To ensure a continuous enclosing contour, we applied morphological closing to fill small discontinuities, followed by a convex hull operation to bridge any remaining gaps.
To validate our method, we compared MRI-derived with manually measured waist and hip circumferences in NAKO participants. MRI-derived values consistently overestimated manual measures (hip: +4.8 cm, waist: +7.8 cm) across 3,572 (hip) and 3,581 (waist) out of the 3,726 individuals.  Agreement with manual measures was high (r = 0.94, p < 0.001) for both waist and hip circumferences.


Supplementary Results
Additional supplementary analyses include stratification by sex and BMI category (Supplementary Table 3 and Figure 2d), association of health behavior indicators on body composition (further detailed below; Supplementary Figure 3), and extended correlation matrices and residual distributions of body composition parameters adjusted for height and BMI (Supplementary Figures 4 and Table 6). Additional outcome analyses include sex-separate model coefficients (Supplementary Figure 5), adjusted body composition stratified by disease (Supplementary Figure 6), and predicted prevalences of clinical conditions not shown in the main results (Supplementary Figure 7). Additional data regarding the discriminative value of body composition beyond anthropometric measures includes the method of waist-to-hip ratio extraction as detailed above (Supplementary Figure 8), receiver operating characteristic (ROC) curves of outcomes not shown in the main results (Supplementary Figure 9), model performance statistics and detailed net reclassification index (NRI) metrics (Supplementary Table 7-9), calibration metrics of all outcomes (Supplementary Figure 10 and Table 10), and decision curve analysis of main outcomes (Supplementary Figure 11 and Table 11). 

Associations of health behavior indicators with body composition
We assessed associations between self-reported alcohol intake frequency and adjusted body composition parameters in 18,974 UKB participants using linear mixed-effects models adjusted for age, height, BMI, and sex (in combined analyses), with imaging center as a random effect. 
Alcohol intake showed inverse associations with subcutaneous fat compartments. Compared to abstainers, participants consuming alcohol 3–4 times per week had lower adjusted ASAT (β = –0.054, 95% CI [–0.080, –0.027], p = 0.0001) and GFAT (β = –0.060, 95% CI [–0.090, –0.030], p = 0.0001). A similar inverse association was observed for daily alcohol intake. In contrast, VAT and liver fat fraction (LFF) were not consistently associated with alcohol frequency.
Notably, alcohol intake frequency was positively associated with adjusted trunk muscle volume, particularly in the 3–4 times per week group (β = 0.093, 95% CI [0.073–0.113], p < 0.0001).
These cross-sectional associations may reflect complex behavioral or health-related confounding. Specifically, individuals who abstain from alcohol may represent a heterogeneous group that includes former drinkers and individuals with underlying health conditions or comorbidities, which could confound associations with muscle mass or adiposity. These findings should be interpreted as descriptive associations only. Alcohol-related results were not adjusted for potential confounders such as diet, education, medication use, or comorbidity burden, limiting interpretability. These exploratory analyses therefore require confirmation in longitudinal cohorts.
Full descriptive statistics for all demographic and clinical variables are provided in Table 1, including sex, age, BMI category, ethnicity, hand grip strength, and physical activity group.


Supplementary Tables
Supplementary Table 1: Additional descriptive statistics of NAKO participants
Dataset	NAKO
Sex	Male  (n = 14,969)	Female  (n = 11,908)
Ethnicity		
White	14,148 (94.52%)	11,050 (92.79%)
African	59 (0.39%)	34 (0.29%)
Indian	24 (0.16%)	10 (0.08%)
Southeast Asian	37 (0.25%)	45 (0.38%)
Northeast Asian	12 (0.08%)	15 (0.13%)
Japanese	4 (0.03%)	5 (0.04%)
South- & Middle American	22 (0.15%)	19 (0.16%)
Not specified	663 (4.43%)	730 (6.13%)


Supplementary Table 2: Additional descriptive statistics of UKB participants 
Dataset	UKB
Sex	Male  (n = 9,094)	Female  (n = 9,880)
Ethnicity		
White	8,779 (96.5%)	9,601 (97.2%)
Black	49 (0.5%)	56 (0.6%)
South Asian	127 (1.4%)	62 (0.6%)
East Asian	25 (0.3%)	38 (0.4%)
Other	114 (1.3%)	123 (1.2%)


Supplementary Table 3: Body composition distribution by sex, age, and BMI category. Table 3 integrates both BMI and age effects on body composition, providing a complete overview of the primary determinants of fat and muscle distribution. The table highlights how body fat compartments differ markedly between sexes, change systematically with aging, and vary across BMI categories. Panel A presents pooled median values and interquartile ranges (IQR) categorized by sex and BMI category based on harmonized data from the UK Biobank (UKB) and German National Cohort (NAKO). Panel B presents median and IQR by sex and age group. Panel C summarizes relative age-related changes and sex-specific ratios, showing percentage changes from early to late adulthood (20-30 years to 60-70 years) and female-to-male ratios in early and late adulthood.
A. Body composition by sex and BMI category
Sex	BMI Category	ASAT (L)	GFAT (L)	VAT (L)	Muscle (L)	LFF (%)	Total SAT (L)
Female	Normal n = 11440	3.15 [2.27, 4.00]	6.74 [5.44, 8.11]	0.83 [0.58, 1.20]	4.27 [3.94, 4.59]	5.90 [4.72, 7.26]	13.49 [10.93, 15.94]
	Overweight n = 6569	5.68 [4.82, 6.61]	9.63 [8.04, 11.17]	1.72 [1.21, 2.35]	4.81 [4.48, 5.15]	7.05 [6.03, 8.88]	20.86 [18.56, 23.25]
	Obese n = 3779	9.00 [7.64, 10.86]	13.07 [11.01, 15.24]	2.96 [2.25, 3.74]	5.54 [5.14, 5.99]	9.17 [7.27, 14.31]	29.79 [26.51, 34.04]
Male	Normal n = 8706	2.46 [1.82, 3.11]	3.37 [2.63, 4.21]	1.50 [0.96, 2.28]	5.66 [5.23, 6.09]	6.56 [5.43, 7.99]	8.57 [6.82, 10.35]
	Overweight n = 10964	4.16 [3.38, 5.07]	4.86 [3.96, 5.89]	3.26 [2.36, 4.19]	6.48 [6.06, 6.93]	8.23 [6.94, 10.92]	13.08 [11.13, 15.26]
	Obese n = 4393	7.02 [5.7, 8.84]	7.37 [6.02, 9.09]	5.21 [4.20, 6.31]	7.45 [6.94, 7.99]	12.03 [8.97, 18.28]	20.10 [17.05, 23.97]
B. Body composition by sex and age group
Female	20-30 years n = 1389	3.43 [2.32, 5.35]	8.35 [6.75, 10.74]	0.58 [0.43, 0.90]	4.66 [4.29, 5.07]	4.55 [3.97, 5.35]	15.11 [11.67, 20.4]
	40-50 years n = 4442	4.31 [2.84, 6.46]	8.74 [6.82, 11.16]	0.99 [0.61, 1.75]	4.77 [4.34, 5.27]	5.72 [4.78, 7.19]	17.52 [13.15, 23.26]
	60-70 years n = 6632	4.71 [3.32, 6.53]	7.92 [6.05, 10.27]	1.62 [1.03, 2.53]	4.47 [4.05, 4.94]	7.52 [6.47, 9.37]	17.62 [13.54, 23.02]
Male	20-30 years n = 1880	2.87 [1.83, 4.53]	4.74 [3.36, 6.46]	1.03 [0.69, 1.82]	6.21 [5.69, 6.80]	5.36 [4.64, 6.52]	10.30 [7.11, 14.74]
	40-50 years n = 5253	4.03 [2.89, 5.68]	4.88 [3.75, 6.38]	2.71 [1.62, 3.99]	6.48 [5.92, 7.12]	7.38 [6.14, 10.41]	12.76 [9.78, 16.66]
	60-70 years n = 6702	3.77 [2.72, 5.09]	4.31 [3.28, 5.70]	3.46 [2.21, 4.78]	6.21 [5.61, 6.85]	8.69 [7.38, 11.82]	12.04 [9.22, 15.44]
C. Age and sex effects
Metric	Age Effect Female	Age Effect Male	Sex Effect Young Adult F/M	Sex Effect Older Adult F/M Ratio
ASAT	1.37	1.31	1.20	1.25
GFAT	0.95	0.91	1.76	1.84
VAT	2.79	3.36	0.56	0.47
Muscle	0.96	1.00	0.75	0.72
LFF	1.65	1.62	0.85	0.87
Total SAT	1.17	1.17	1.47	1.46

Note: Values in Panel A and B represent median volumes and interquartile ranges (IQR). In Panel C, age effects represent relative changes from age 20-30 to age 60-70, and sex effects represent female-to-male ratios in the 20-30 and 60-70 age group respectively. ASAT = abdominal subcutaneous adipose tissue; GFAT = gluteofemoral adipose tissue; VAT = visceral adipose tissue; LFF = liver fat fraction; Total SAT = total subcutaneous adipose tissue.


Supplementary Table 4. NAKO Linear Mixed-Effects Model Coefficient Estimates
Response Variable  (Z-Score)	ASAT	GFAT	VAT	Muscle	LFF
Predictors	Estimates	p	Estimates	p	Estimates	p	Estimates	p	Estimates	p
(Intercept)	0.346	<0.0001	0.765	<0.0001	-0.248	<0.0001	-0.287	<0.0001	-0.186	<0.0001
	(0.325 - 0.367)		(0.711 - 0.818)		(-0.279 - -0.217)		(-0.316 - -0.257)		(-0.234 - -0.138)	
Standardized BMI	0.927	<0.0001	0.648	<0.0001	0.616	<0.0001	0.522	<0.0001	0.496	<0.0001
	(0.922 - 0.932)		(0.643 - 0.654)		(0.609 - 0.623)		(0.519 - 0.526)		(0.486 - 0.506)	
Sex [Male]	-0.577	<0.0001	-1.384	<0.0001	0.685	<0.0001	0.503	<0.0001	0.476	<0.0001
	(-0.594 - -0.560)		(-1.403 - -1.365)		(0.662 - 0.708)		(0.490 - 0.516)		(0.443 - 0.508)	
Standardized Age	-0.042	<0.0001	-0.136	<0.0001	0.275	<0.0001	-0.033	<0.0001	0.179	<0.0001
	(-0.048 - -0.037)		(-0.142 - -0.130)		(0.268 - 0.283)		(-0.037 - -0.029)		(0.168 - 0.190)	
Standardized Height	0.248	<0.0001	0.199	<0.0001	0.174	<0.0001	0.388	<0.0001		
	(0.241 - 0.256)		(0.190 - 0.208)		(0.163 - 0.184)		(0.382 - 0.394)			
Activity group [Sufficient]	-0.028	0.0183	-0.025	0.0659	-0.037	0.0239	0.019	0.0373	-0.01	0.6716
	(-0.052 - -0.005)		(-0.051 - 0.002)		(-0.069 - -0.005)		(0.001 - 0.037)		(-0.059 - 0.038)	
Activity group [Moderate]	-0.065	<0.0001	-0.052	<0.0001	-0.13	<0.0001	0.032	<0.0001	-0.099	<0.0001
	(-0.082 - -0.049)		(-0.070 - -0.034)		(-0.152 - -0.107)		(0.019 - 0.044)		(-0.132 - -0.065)	
Activity group [High]	-0.096	<0.0001	-0.105	<0.0001	-0.156	<0.0001	0.053	<0.0001	-0.13	<0.0001
	(-0.111 - -0.081)		(-0.121 - -0.088)		(-0.176 - -0.136)		(0.042 - 0.065)		(-0.160 - -0.100)	
Standardized       Handgrip strength	-0.162	<0.0001	-0.146	<0.0001	-0.097	<0.0001	0.117	<0.0001	-0.092	<0.0001
	(-0.171 - -0.154)		(-0.156 - -0.137)		(-0.108 - -0.085)		(0.110 - 0.123)		(-0.109 - -0.076)	
Observations	26877	26877	26877	26877	26877
Note: Estimates represent standardized β coefficients with 95% confidence intervals, showing the change in body composition parameter (in standard deviation units) per unit change in the predictor. For categorical variables, effects represent the difference compared to the reference group (sex: female; activity group: insufficient). P-values were derived from Wald z-statistics for fixed effects and adjusted for false discovery using the Benjamini-Hochberg method (α = 0.05). For categorical predictors, each non-reference level is compared to the reference group, with coefficients interpreted as contrasts. * p<0.05, ** p<0.01, *** p<0.001.


Supplementary Table 5. UKB Linear Mixed-Effects Model Coefficient Estimates
Response Variable  (Z-Score)	ASAT	GFAT	VAT	Muscle	LFF
Predictors	Estimates	p	Estimates	p	Estimates	p	Estimates	p	Estimates	p
(Intercept)	0.491	<0.0001	0.861	<0.0001	-0.246	<0.0001	-0.351	<0.0001	-0.006	0.8955
	(0.465 - 0.518)		(0.820 - 0.902)		(-0.296 - -0.197)		(-0.371 - -0.331)		(-0.057 - 0.045)	
Standardized BMI	0.869	<0.0001	0.737	<0.0001	0.649	<0.0001	0.613	<0.0001	0.35	<0.0001
	(0.863 - 0.876)		(0.730 - 0.744)		(0.640 - 0.658)		(0.608 - 0.618)		(0.337 - 0.363)	
Sex [Male]	-0.864	<0.0001	-1.617	<0.0001	0.551	<0.0001	0.624	<0.0001	0.156	<0.0001
	(-0.883 - -0.846)		(-1.638 - -1.597)		(0.527 - 0.576)		(0.610 - 0.638)		(0.131 - 0.181)	
Standardized Age	-0.022	0.0001	-0.028	<0.0001	0.144	<0.0001	-0.103	<0.0001	0.086	<0.0001
	(-0.032 - -0.011)		(-0.040 - -0.016)		(0.130 - 0.159)		(-0.111 - -0.095)		(0.065 - 0.107)	
Standardized Height	0.206	<0.0001	0.267	<0.0001	0.162	<0.0001	0.525	<0.0001		
	(0.197 - 0.215)		(0.257 - 0.277)		(0.149 - 0.174)		(0.518 - 0.531)			
Alcohol [Special occasions]	0.004	0.8056	-0.016	0.3437	-0.001	0.9767	0.01	0.4676	0.003	0.9293
	(-0.027 - 0.034)		(-0.050 - 0.018)		(-0.041 - 0.040)		(-0.013 - 0.033)		(-0.058 - 0.063)	
Alcohol [1to3pm]	-0.004	0.8056	-0.028	0.1009	0.045	0.0401	0.059	<0.0001	0.01	0.8948
	(-0.034 - 0.025)		(-0.062 - 0.005)		(0.005 - 0.085)		(0.036 - 0.081)		(-0.049 - 0.069)	
Alcohol [1to2pw]	-0.035	0.0125	-0.041	0.0076	0.025	0.2059	0.078	<0.0001	-0.078	0.0105
	(-0.061 - -0.008)		(-0.071 - -0.012)		(-0.011 - 0.060)		(0.058 - 0.098)		(-0.131 - -0.025)	
Alcohol [3to4pw]	-0.054	0.0001	-0.06	0.0001	0.018	0.3612	0.093	<0.0001	-0.069	0.0194
	(-0.080 - -0.027)		(-0.090 - -0.030)		(-0.018 - 0.053)		(0.073 - 0.113)		(-0.122 - -0.016)	
Alcohol [Daily]	-0.065	<0.0001	-0.081	<0.0001	0.055	0.0073	0.087	<0.0001	0.017	0.7777
	(-0.093 - -0.037)		(-0.112 - -0.049)		(0.017 - 0.093)		(0.066 - 0.109)		(-0.040 - 0.073)	
Smoking [Previous]	-0.02	0.0033	-0.029	0.0002	0.048	<0.0001	0.002	0.7066	0.031	0.0318
	(-0.034 - -0.007)		(-0.044 - -0.014)		(0.031 - 0.066)		(-0.008 - 0.012)		(0.005 - 0.057)	
Smoking [Current]	-0.052	0.0019	-0.072	0.0001	0.046	0.0426	-0.008	0.5551	0.085	0.0183
	(-0.083 - -0.020)		(-0.107 - -0.036)		(0.004 - 0.089)		(-0.032 - 0.016)		(0.022 - 0.148)	
Observations	18974	18974	18974	18974	18974
Note: Estimates represent standardized β coefficients with 95% confidence intervals, showing the change in body composition parameter (in standard deviation units) per unit change in the predictor. For categorical variables, effects represent the difference compared to the reference group (sex: female; alcohol: never; smoking: never). P-values were derived from Wald z-statistics for fixed effects and adjusted for false discovery using the Benjamini-Hochberg method (α = 0.05). For categorical predictors, each non-reference level is compared to the reference group, with coefficients interpreted as contrasts. * p<0.05, ** p<0.01, *** p<0.001.


Supplementary Table 6: Pearson correlation coefficients between body composition parameters
Parameter 1	Parameter 2	Male	Female
ASAT adj.	GFAT adj.	0.565 [0.556, 0.573]	0.049 [0.036, 0.063]
ASAT adj.	VAT adj.	0.098 [0.085, 0.110]	0.275 [0.263, 0.287]
ASAT adj.	Muscle adj.	-0.398 [-0.409, -0.388]	-0.132 [-0.145, -0.119]
ASAT adj.	LFF adj.	0.040 [0.027, 0.052]	0.114 [0.101, 0.127]
GFAT adj.	VAT adj.	-0.103 [-0.116, -0.091]	-0.337 [-0.349, -0.325]
GFAT adj.	Muscle adj.	-0.407 [-0.418, -0.397]	-0.376 [-0.388, -0.365]
GFAT adj.	LFF adj.	-0.088 [-0.100, -0.075]	-0.233 [-0.245, -0.22]
VAT adj.	Muscle adj.	0.115 [0.102, 0.127]	0.257 [0.245, 0.27]
VAT adj.	LFF adj.	0.350 [0.339, 0.361]	0.427 [0.416, 0.438]
Muscle adj.	LFF adj.	0.048 [0.035, 0.061]	0.119 [0.106, 0.132]

Note: Values represent Pearson correlation coefficients and 95% confidence intervals between adjusted Z-scores of body composition parameters. ASAT = abdominal subcutaneous adipose tissue; GFAT = gluteofemoral adipose tissue; VAT = visceral adipose tissue; LFF = liver fat fraction; adj. = adjusted. 


Supplementary Table 7: Model-comparison statistics for males. For pooled non-cancer outcomes, the complete-case analysis included n = 23,651 men; cancer was restricted to UKB men (n = 9,094). Shown are likelihood-ratio test (LRT) results, area under the curve (AUC), change in AUC (ΔAUC, DeLong’s test), and continuous net reclassification improvement (cNRI, Wald test) for generalized linear mixed-effects models with incremental adjustment for anthropometric variables and adjusted body-composition parameters (adj. BCPs). 95% confidence intervals are shown in parentheses. All P values were adjusted using the Benjamini–Hochberg (BH) method. LRTs test improvement in model fit; higher AUC and cNRI values indicate better discrimination and reclassification, respectively.
Outcome	Model	LRT χ2	LRT p-adj	AUC	ΔAUC	ΔAUC p-adj	cNRI	NRI p-adj
Cancer	Base			0.6665 (0.6494-0.6836)				
Cancer	Base + BMI	0.018	0.8920728	0.6663 (0.6492-0.6834)	-0.0002 (-0.0010-0.0005)	0.6309862	0.0040 (-0.0630-0.0690)	0.9085913
Cancer	Base + BMI + WHR	0.501	0.5390001	0.6666 (0.6495-0.6836)	0.0003 (-0.0007-0.0013)	0.6309862	0.0040 (-0.0320-0.0440)	0.8447312
Cancer	Base + BMI + WHR + adj. BCPs	7.009	0.2700085	0.6685 (0.6514-0.6856)	0.0019 (-0.0010-0.0049)	0.2313428	0.0620 (-0.0030-0.1260)	0.075077
Cancer	Base + BCPs			0.6676 (0.6505-0.6847)	-0.0009 (-0.0033-0.0015)	0.5412998	-0.0340 (-0.0970-0.0290)	0.3354682
Hypertension	Base			0.6663 (0.6590-0.6737)				
Hypertension	Base + BMI	1257.172	<0.0001	0.7262 (0.7192-0.7333)	0.0599 (0.0552-0.0646)	<0.0001	0.4750 (0.4450-0.5030)	<0.0001
Hypertension	Base + BMI + WHR	198.021	<0.0001	0.7378 (0.7309-0.7447)	0.0116 (0.0097-0.0134)	<0.0001	0.1920 (0.1650-0.2220)	<0.0001
Hypertension	Base + BMI + WHR + adj. BCPs	106.892	<0.0001	0.7390 (0.7320-0.7459)	0.0011 (-0.0001-0.0024)	0.1020521	0.0890 (0.0620-0.1180)	<0.0001
Hypertension	Base + BCPs			0.7323 (0.7253-0.7393)	-0.0066 (-0.0081--0.0051)	<0.0001	-0.1690 (-0.1990--0.1410)	<0.0001
CAD	Base			0.7829 (0.7674-0.7983)				
CAD	Base + BMI	52.188	<0.0001	0.7908 (0.7756-0.8061)	0.0080 (0.0026-0.0134)	0.0058304	0.2390 (0.1650-0.3150)	<0.0001
CAD	Base + BMI + WHR	11.937	0.0009285	0.7911 (0.7760-0.8062)	0.0003 (-0.0024-0.0030)	0.8549614	0.0450 (-0.0310-0.1230)	0.2870746
CAD	Base + BMI + WHR + adj. BCPs	17.186	0.0059124	0.7946 (0.7795-0.8097)	0.0035 (0.0006-0.0064)	0.026196	0.2140 (0.1420-0.2950)	<0.0001
CAD	Base + BCPs			0.7916 (0.7766-0.8066)	-0.0030 (-0.0058--0.0002)	0.0458294	-0.1410 (-0.2160--0.0650)	0.0006106
Heart failure	Base			0.5683 (0.5326-0.6039)				
Heart failure	Base + BMI	2.372	0.1588523	0.5787 (0.5420-0.6154)	0.0104 (0.0039-0.0169)	0.0026357	0.1780 (0.0450-0.3180)	0.017669
Heart failure	Base + BMI + WHR	0.101	0.8102035	0.5813 (0.5446-0.6180)	0.0026 (0.0012-0.0040)	0.0004817	0.1960 (0.0530-0.3410)	0.0124055
Heart failure	Base + BMI + WHR + adj. BCPs	12.057	0.0459278	0.5975 (0.5601-0.6349)	0.0162 (0.0028-0.0296)	0.0257049	0.1220 (-0.0350-0.2700)	0.137003
Heart failure	Base + BCPs			0.5971 (0.5595-0.6347)	-0.0004 (-0.0062-0.0054)	0.8890068	-0.0740 (-0.2200-0.0840)	0.392458
Diabetes	Base			0.6885 (0.6762-0.7008)				
Diabetes	Base + BMI	479.137	<0.0001	0.7564 (0.7440-0.7687)	0.0679 (0.0584-0.0773)	<0.0001	0.5380 (0.4860-0.5960)	<0.0001
Diabetes	Base + BMI + WHR	310.771	<0.0001	0.7877 (0.7756-0.7999)	0.0313 (0.0248-0.0378)	<0.0001	0.4480 (0.3930-0.5000)	<0.0001
Diabetes	Base + BMI + WHR + adj. BCPs	266.086	<0.0001	0.8054 (0.7935-0.8173)	0.0177 (0.0127-0.0227)	<0.0001	0.3480 (0.2900-0.4030)	<0.0001
Diabetes	Base + BCPs			0.7919 (0.7797-0.8040)	-0.0135 (-0.0183--0.0087)	<0.0001	-0.3520 (-0.4100--0.2960)	<0.0001
Hyperlipidemia	Base			0.6029 (0.5957-0.6101)				
Hyperlipidemia	Base + BMI	848.264	<0.0001	0.6530 (0.6461-0.6599)	0.0501 (0.0450-0.0551)	<0.0001	0.3310 (0.3060-0.3550)	<0.0001
Hyperlipidemia	Base + BMI + WHR	454.543	<0.0001	0.6758 (0.6690-0.6826)	0.0228 (0.0194-0.0262)	<0.0001	0.2160 (0.1900-0.2400)	<0.0001
Hyperlipidemia	Base + BMI + WHR + adj. BCPs	368.003	<0.0001	0.6921 (0.6854-0.6987)	0.0163 (0.0136-0.0189)	<0.0001	0.2100 (0.1870-0.2340)	<0.0001
Hyperlipidemia	Base + BCPs			0.6885 (0.6818-0.6952)	-0.0036 (-0.0049--0.0022)	<0.0001	-0.1050 (-0.1310--0.0800)	<0.0001
Gout	Base			0.6130 (0.5983-0.6276)				
Gout	Base + BMI	288.139	<0.0001	0.6936 (0.6784-0.7089)	0.0806 (0.0695-0.0918)	<0.0001	0.5020 (0.4420-0.5680)	<0.0001
Gout	Base + BMI + WHR	32.169	<0.0001	0.7086 (0.6936-0.7235)	0.0149 (0.0114-0.0185)	<0.0001	0.2470 (0.1880-0.3140)	<0.0001
Gout	Base + BMI + WHR + adj. BCPs	46.072	<0.0001	0.7149 (0.6999-0.7300)	0.0064 (0.0027-0.0101)	0.0013636	0.1770 (0.1140-0.2370)	<0.0001
Gout	Base + BCPs			0.7061 (0.6909-0.7214)	-0.0088 (-0.0121--0.0055)	<0.0001	-0.1520 (-0.2150--0.0900)	<0.0001
Back pain	Base			0.5494 (0.5397-0.5592)				
Back pain	Base + BMI	9.950	0.0024132	0.5417 (0.5318-0.5517)	-0.0077 (-0.0096--0.0058)	<0.0001	0.1410 (0.1030-0.1780)	<0.0001
Back pain	Base + BMI + WHR	1.370	0.2838343	0.5379 (0.5279-0.5479)	-0.0038 (-0.0046--0.0031)	<0.0001	0.1800 (0.1420-0.2180)	<0.0001
Back pain	Base + BMI + WHR + adj. BCPs	31.334	<0.0001	0.5313 (0.5212-0.5414)	-0.0066 (-0.0097--0.0035)	<0.0001	0.0060 (-0.0330-0.0450)	0.8025362
Back pain	Base + BCPs			0.5400 (0.5300-0.5500)	0.0087 (0.0056-0.0118)	<0.0001	-0.0160 (-0.0530-0.0220)	0.4390232
Osteoporosis	Base			0.5881 (0.5559-0.6204)				
Osteoporosis	Base + BMI	0.033	0.8898626	0.5891 (0.5567-0.6215)	0.0010 (-0.0002-0.0021)	0.1293995	0.1440 (-0.0090-0.2870)	0.0666142
Osteoporosis	Base + BMI + WHR	16.942	<0.0001	0.6359 (0.6022-0.6696)	0.0468 (0.0280-0.0655)	<0.0001	0.3950 (0.2480-0.5320)	<0.0001
Osteoporosis	Base + BMI + WHR + adj. BCPs	20.297	0.0017458	0.6596 (0.6267-0.6925)	0.0237 (0.0051-0.0424)	0.019072	0.1470 (-0.0020-0.2970)	0.0666142
Osteoporosis	Base + BCPs			0.6759 (0.6429-0.7090)	0.0163 (0.0004-0.0322)	0.0568002	0.1450 (0.0010-0.2900)	0.0666142


Supplementary Table 8: Model-comparison statistics for females. For pooled non-cancer outcomes, the complete-case analysis included n = 21,587 women; cancer was restricted to UKB women (n = 9,880). Shown are likelihood-ratio test (LRT) results, area under the curve (AUC), change in AUC (ΔAUC, DeLong’s test), and continuous net reclassification improvement (cNRI, Wald test) for generalized linear mixed-effects models with incremental adjustment for anthropometric variables and adjusted body-composition parameters (adj. BCPs). 95% confidence intervals are shown in parentheses. All P values were adjusted using the Benjamini–Hochberg (BH) method. LRTs test improvement in model fit; higher AUC and cNRI values indicate better discrimination and reclassification, respectively.
Outcome	Model	LRT χ2	LRT p-adj	AUC	ΔAUC	ΔAUC p-adj	cNRI	NRI p-adj
Cancer	Base			0.5715 (0.5542-0.5888)				
Cancer	Base + BMI	0.073	0.7868328	0.5716 (0.5543-0.5889)	0.0001 (-0.0006-0.0008)	0.7892216	0.0170 (-0.0430-0.0760)	0.6067533
Cancer	Base + BMI + WHR	1.748	0.1933303	0.5726 (0.5553-0.5900)	0.0010 (-0.0018-0.0039)	0.5370293	0.0270 (-0.0350-0.0900)	0.4496409
Cancer	Base + BMI + WHR + adj. BCPs	23.045	0.0005956	0.5811 (0.5639-0.5983)	0.0085 (-0.0001-0.0170)	0.0691185	0.0880 (0.0280-0.1500)	0.0075091
Cancer	Base + BCPs			0.5801 (0.5628-0.5973)	-0.0011 (-0.0063-0.0042)	0.7310353	0.0120 (-0.0500-0.0770)	0.7421137
Hypertension	Base			0.6549 (0.6463-0.6634)				
Hypertension	Base + BMI	1191.432	<0.0001	0.7294 (0.7214-0.7375)	0.0746 (0.0686-0.0806)	<0.0001	0.5350 (0.5020-0.5690)	<0.0001
Hypertension	Base + BMI + WHR	148.832	<0.0001	0.7372 (0.7292-0.7451)	0.0077 (0.0057-0.0098)	<0.0001	0.2100 (0.1760-0.2430)	<0.0001
Hypertension	Base + BMI + WHR + adj. BCPs	122.438	<0.0001	0.7415 (0.7336-0.7495)	0.0044 (0.0027-0.0060)	<0.0001	0.0460 (0.0140-0.0800)	0.0099546
Hypertension	Base + BCPs			0.7329 (0.7249-0.7409)	-0.0087 (-0.0102--0.0072)	<0.0001	-0.1970 (-0.2300--0.1610)	<0.0001
CAD	Base			0.7229 (0.6919-0.7540)				
CAD	Base + BMI	47.518	<0.0001	0.7543 (0.7253-0.7833)	0.0314 (0.0146-0.0481)	0.0004961	0.3570 (0.2290-0.4850)	<0.0001
CAD	Base + BMI + WHR	9.850	0.0025472	0.7594 (0.7304-0.7884)	0.0051 (-0.0021-0.0123)	0.1895356	0.2260 (0.0970-0.3600)	0.0010766
CAD	Base + BMI + WHR + adj. BCPs	10.986	0.0581063	0.7670 (0.7383-0.7958)	0.0076 (0.0013-0.0139)	0.027435	0.0370 (-0.0950-0.1640)	0.6067533
CAD	Base + BCPs			0.7660 (0.7373-0.7948)	-0.0010 (-0.0084-0.0064)	0.7892216	0.0650 (-0.0560-0.1890)	0.3389697
Heart failure	Base			0.5070 (0.4649-0.5491)				
Heart failure	Base + BMI	6.438	0.0142672	0.5273 (0.4823-0.5723)	0.0203 (-0.0646-0.1052)	0.6975078	0.2410 (0.0900-0.4180)	0.0040128
Heart failure	Base + BMI + WHR	8.280	0.0054101	0.5593 (0.5130-0.6057)	0.0321 (0.0129-0.0513)	0.0018201	0.1940 (0.0470-0.3430)	0.0167495
Heart failure	Base + BMI + WHR + adj. BCPs	14.721	0.0142672	0.5794 (0.5326-0.6261)	0.0200 (-0.0013-0.0414)	0.0847179	0.1820 (0.0240-0.3300)	0.0294094
Heart failure	Base + BCPs			0.5457 (0.4986-0.5929)	-0.0336 (-0.0460--0.0213)	<0.0001	-0.3310 (-0.4760--0.1830)	<0.0001
Diabetes	Base			0.5902 (0.5710-0.6093)				
Diabetes	Base + BMI	395.225	<0.0001	0.7126 (0.6938-0.7313)	0.1224 (0.1048-0.1400)	<0.0001	0.6100 (0.5380-0.6780)	<0.0001
Diabetes	Base + BMI + WHR	267.747	<0.0001	0.7504 (0.7321-0.7688)	0.0379 (0.0276-0.0482)	<0.0001	0.5110 (0.4430-0.5780)	<0.0001
Diabetes	Base + BMI + WHR + adj. BCPs	225.025	<0.0001	0.7688 (0.7500-0.7876)	0.0184 (0.0115-0.0252)	<0.0001	0.1680 (0.0990-0.2410)	<0.0001
Diabetes	Base + BCPs			0.7595 (0.7404-0.7785)	-0.0093 (-0.0148--0.0039)	0.0013248	-0.3190 (-0.3920--0.2530)	<0.0001
Hyperlipidemia	Base			0.6892 (0.6821-0.6963)				
Hyperlipidemia	Base + BMI	266.337	<0.0001	0.7010 (0.6940-0.7079)	0.0118 (0.0095-0.0140)	<0.0001	0.2260 (0.1990-0.2530)	<0.0001
Hyperlipidemia	Base + BMI + WHR	240.443	<0.0001	0.7107 (0.7038-0.7175)	0.0097 (0.0077-0.0118)	<0.0001	0.2160 (0.1880-0.2420)	<0.0001
Hyperlipidemia	Base + BMI + WHR + adj. BCPs	303.492	<0.0001	0.7210 (0.7142-0.7278)	0.0103 (0.0082-0.0124)	<0.0001	0.1750 (0.1490-0.2020)	<0.0001
Hyperlipidemia	Base + BCPs			0.7176 (0.7108-0.7244)	-0.0034 (-0.0040--0.0027)	<0.0001	0.0820 (0.0550-0.1090)	<0.0001
Gout	Base			0.6007 (0.5668-0.6347)				
Gout	Base + BMI	97.635	<0.0001	0.7159 (0.6820-0.7499)	0.1152 (0.0897-0.1407)	<0.0001	0.6760 (0.5440-0.8070)	<0.0001
Gout	Base + BMI + WHR	5.975	0.0170348	0.7249 (0.6908-0.7589)	0.0090 (0.0025-0.0154)	0.0104204	0.2750 (0.1400-0.4080)	0.0001511
Gout	Base + BMI + WHR + adj. BCPs	17.686	0.0047853	0.7318 (0.6973-0.7662)	0.0069 (-0.0018-0.0156)	0.1447532	0.0810 (-0.0560-0.2240)	0.3002971
Gout	Base + BCPs			0.7260 (0.6912-0.7607)	-0.0058 (-0.0091--0.0024)	0.0013248	-0.2230 (-0.3600--0.0880)	0.0016816
Back pain	Base			0.5743 (0.5641-0.5845)				
Back pain	Base + BMI	49.976	<0.0001	0.5408 (0.5299-0.5517)	-0.0335 (-0.0383--0.0287)	<0.0001	0.2020 (0.1630-0.2410)	<0.0001
Back pain	Base + BMI + WHR	10.969	0.0014714	0.5319 (0.5209-0.5429)	-0.0089 (-0.0111--0.0066)	<0.0001	0.1060 (0.0650-0.1410)	<0.0001
Back pain	Base + BMI + WHR + adj. BCPs	21.749	0.0009861	0.5261 (0.5149-0.5372)	-0.0059 (-0.0086--0.0031)	<0.0001	0.0250 (-0.0110-0.0640)	0.2343666
Back pain	Base + BCPs			0.5400 (0.5290-0.5510)	0.0140 (0.0108-0.0171)	<0.0001	-0.1010 (-0.1400--0.0620)	<0.0001
Osteoporosis	Base			0.7048 (0.6884-0.7212)				
Osteoporosis	Base + BMI	41.389	<0.0001	0.7091 (0.6922-0.7260)	0.0043 (-0.0010-0.0097)	0.1393776	0.1650 (0.0970-0.2400)	<0.0001
Osteoporosis	Base + BMI + WHR	2.536	0.1201966	0.7104 (0.6935-0.7272)	0.0013 (0.0001-0.0025)	0.0482625	0.1060 (0.0270-0.1800)	0.0089051
Osteoporosis	Base + BMI + WHR + adj. BCPs	27.639	<0.0001	0.7163 (0.6996-0.7330)	0.0059 (0.0020-0.0097)	0.0045086	0.1520 (0.0780-0.2240)	<0.0001
Osteoporosis	Base + BCPs			0.7114 (0.6947-0.7280)	-0.0049 (-0.0092--0.0006)	0.0386037	-0.0100 (-0.0800-0.0700)	0.7903727


Supplementary Table 9: Continuous net reclassification improvement (cNRI) and its components comparing generalized linear mixed-effects models with and without additional adjustment for body-composition parameters (BCPs), presented separately for males and females. Shown are cNRI values and 95% confidence intervals, as well as NRI for events (NRI+), NRI for non-events (NRI−), the probability of upward and downward reclassification for cases (Pr(Up|Case), Pr(Down|Case)), and for controls (Pr(Down|Ctrl), Pr(Up|Ctrl)). The cNRI estimates and confidence intervals are the same final model-comparison results reported in Supplementary Tables 7 and 8; the component metrics are shown to describe the direction of reclassification.
Outcome	Sex	NRI	NRI+	NRI-	Pr (Up|Case)	Pr (Down|Case)	Pr (Down|Ctrl)	Pr (Up|Ctrl)
Cancer	Male	0.062 [-0.003, 0.126]	0.098 [0.032, 0.161]	-0.036 [-0.057, -0.016]	0.549 [0.516, 0.581]	0.451 [0.419, 0.484]	0.482 [0.472, 0.492]	0.518 [0.508, 0.528]
	Female	0.088 [0.028, 0.150]	-0.022 [-0.081, 0.03]	0.11 [0.089, 0.132]	0.489 [0.459, 0.515]	0.511 [0.485, 0.541]	0.555 [0.544, 0.566]	0.445 [0.434, 0.456]
Hyper-tension	Male	0.089 [0.062, 0.118]	0.161 [0.135, 0.185]	-0.072 [-0.088, -0.057]	0.581 [0.567, 0.593]	0.419 [0.407, 0.433]	0.464 [0.456, 0.472]	0.536 [0.528, 0.544]
	Female	0.046 [0.014, 0.080]	0.05 [0.02, 0.079]	-0.004 [-0.019, 0.012]	0.525 [0.51, 0.539]	0.475 [0.461, 0.49]	0.498 [0.49, 0.506]	0.502 [0.494, 0.51]
CAD	Male	0.214 [0.142, 0.295]	0.186 [0.113, 0.262]	0.028 [0.014, 0.04]	0.593 [0.556, 0.631]	0.407 [0.369, 0.444]	0.514 [0.507, 0.52]	0.486 [0.48, 0.493]
	Female	0.037 [-0.095, 0.164]	-0.117 [-0.247, 0.018]	0.153 [0.139, 0.167]	0.442 [0.376, 0.509]	0.558 [0.491, 0.624]	0.577 [0.57, 0.584]	0.423 [0.416, 0.43]
Heart failure	Male	0.122 [-0.035, 0.270]	0.016 [-0.139, 0.161]	0.106 [0.092, 0.119]	0.508 [0.431, 0.58]	0.492 [0.42, 0.569]	0.553 [0.546, 0.559]	0.447 [0.441, 0.454]
	Female	0.182 [0.024, 0.330]	0.096 [-0.073, 0.247]	0.086 [0.072, 0.099]	0.548 [0.464, 0.624]	0.452 [0.376, 0.536]	0.543 [0.536, 0.55]	0.457 [0.45, 0.464]
Diabetes	Male	0.348 [0.290, 0.403]	0.089 [0.035, 0.139]	0.26 [0.248, 0.273]	0.544 [0.518, 0.57]	0.456 [0.43, 0.482]	0.63 [0.624, 0.636]	0.37 [0.364, 0.376]
	Female	0.168 [0.099, 0.241]	0.118 [0.055, 0.188]	0.05 [0.037, 0.064]	0.559 [0.528, 0.594]	0.441 [0.406, 0.472]	0.525 [0.518, 0.532]	0.475 [0.468, 0.482]
Hyper-lipidemia	Male	0.210 [0.187, 0.234]	0.176 [0.158, 0.193]	0.034 [0.017, 0.053]	0.588 [0.579, 0.597]	0.412 [0.403, 0.421]	0.517 [0.508, 0.526]	0.483 [0.474, 0.492]
	Female	0.175 [0.149, 0.202]	0.118 [0.097, 0.138]	0.057 [0.038, 0.075]	0.559 [0.548, 0.569]	0.441 [0.431, 0.452]	0.529 [0.519, 0.537]	0.471 [0.463, 0.481]
Gout	Male	0.177 [0.114, 0.237]	0.043 [-0.02, 0.104]	0.134 [0.121, 0.147]	0.521 [0.49, 0.552]	0.479 [0.448, 0.51]	0.567 [0.561, 0.574]	0.433 [0.426, 0.439]
	Female	0.081 [-0.056, 0.224]	0.028 [-0.11, 0.168]	0.053 [0.04, 0.067]	0.514 [0.445, 0.584]	0.486 [0.416, 0.555]	0.526 [0.52, 0.533]	0.474 [0.467, 0.48]
Back pain	Male	0.006 [-0.033, 0.045]	0.037 [0.002, 0.073]	-0.031 [-0.045, -0.017]	0.519 [0.501, 0.537]	0.481 [0.463, 0.499]	0.484 [0.478, 0.491]	0.516 [0.509, 0.522]
	Female	0.025 [-0.011, 0.064]	0.03 [-0.008, 0.066]	-0.004 [-0.019, 0.01]	0.515 [0.496, 0.533]	0.485 [0.467, 0.504]	0.498 [0.491, 0.505]	0.502 [0.495, 0.509]
Osteo-porosis	Male	0.147 [-0.002, 0.297]	0.059 [-0.087, 0.193]	0.087 [0.074, 0.1]	0.53 [0.457, 0.596]	0.47 [0.404, 0.543]	0.544 [0.537, 0.55]	0.456 [0.45, 0.463]
	Female	0.152 [0.078, 0.224]	0.086 [0.014, 0.156]	0.065 [0.052, 0.079]	0.543 [0.507, 0.578]	0.457 [0.422, 0.493]	0.533 [0.526, 0.54]	0.467 [0.46, 0.474]


Supplementary Table 10: Calibration metrics for generalized linear mixed-effects models of all analyzed outcomes in male and female participants. Models include age, BMI, and WHR, with and without adjusted body composition parameters (adjBCPs). Intercept values near zero and slope values near one indicate good calibration, while lower Brier scores reflect better predictive accuracy.
Outcome	Sex	Model	Intercept	Slope	Brier
CAD	M	BMI+WHR	0.042348	1.013553	0.026128
		BMI+WHR+adjBCPs	0.039922	1.012787	0.02607
	F	BMI+WHR	0.04161	1.009609	0.010455
		BMI+WHR+adjBCPs	0.046956	1.010872	0.010449
Type 2 Diabetes	M	BMI+WHR	0.012496	1.004963	0.048441
		BMI+WHR+adjBCPs	0.009607	1.003846	0.047148
	F	BMI+WHR	0.027598	1.009557	0.035558
		BMI+WHR+adjBCPs	0.017977	1.006185	0.034538
Hyperlipidemia	M	BMI+WHR	0.000248	1.004573	0.224929
		BMI+WHR+adjBCPs	0.000275	1.004265	0.220977
	F	BMI+WHR	0.001337	1.004383	0.207491
		BMI+WHR+adjBCPs	0.001267	1.003984	0.204228
Back pain	M	BMI+WHR	0.018139	1.011373	0.100674
		BMI+WHR+adjBCPs	0.017681	1.011107	0.100482
	F	BMI+WHR	0.012313	1.0091	0.104339
		BMI+WHR+adjBCPs	0.012142	1.008974	0.104126
Cancer	M	BMI+WHR	-2.7E-09	1.000000	0.092144
		BMI+WHR+adjBCPs	-2.7E-09	1.000000	0.092049
	F	BMI+WHR	2.39E-13	1.000000	0.10383
		BMI+WHR+adjBCPs	-5.3E-14	1.000000	0.103585
Gout	M	BMI+WHR	0.04474	1.015997	0.038881
		BMI+WHR+adjBCPs	0.041923	1.01508	0.038752
	F	BMI+WHR	0.135105	1.035215	0.009417
		BMI+WHR+adjBCPs	0.123056	1.032225	0.009361
Heart failure	M	BMI+WHR	0.360023	1.083513	0.007668
		BMI+WHR+adjBCPs	0.326557	1.076208	0.007655
	F	BMI+WHR	0.429513	1.098398	0.007548
		BMI+WHR+adjBCPs	0.357428	1.082408	0.007523
Hypertension	M	BMI+WHR	0.003356	1.003897	0.164907
		BMI+WHR+adjBCPs	0.003309	1.003835	0.163932
	F	BMI+WHR	0.005328	1.004525	0.13626
		BMI+WHR+adjBCPs	0.005081	1.00427	0.135151
Osteoporosis	M	BMI+WHR	0.32661	1.072813	0.007685
		BMI+WHR+adjBCPs	0.286195	1.064446	0.007675
	F	BMI+WHR	0.068918	1.022503	0.029674
		BMI+WHR+adjBCPs	0.065937	1.021649	0.029625


Supplementary Table 11: Decision curve analysis based metrics of true positives and false positives per 1,000 participants of models with and without adjusted body composition parameters (adjBCPs) at different probability thresholds corresponding to approximate disease prevalence in the given cohorts. Values in round brackets indicate 95% confidence intervals.
Outcome	Sex	Model	Threshold	TP / 1,000 (95% CI)	FP / 1,000 (95% CI)
CAD	Male	BMI+WHR	0.03	21.82 (20.89 - 22.59)	307.72 (301.54 - 312.93)
		BMI+WHR+adjBCPs	0.03	21.61 (20.55 - 22.5)	305.31 (299.05 - 310.29)
		BMI+WHR	0.04	19.28 (18.3 - 20.24)	231.66 (226.19 - 237.33)
		BMI+WHR+adjBCPs	0.04	19.11 (18.15 - 20.54)	231.45 (224.62 - 236.63)
		BMI+WHR	0.05	16.53 (15.61 - 17.43)	175.43 (170.61 - 180.58)
		BMI+WHR+adjBCPs	0.05	16.79 (15.81 - 18.19)	176.06 (171.65 - 180.8)
		BMI+WHR	0.1	5.12 (4.16 - 5.8)	39.28 (36.08 - 42.11)
		BMI+WHR+adjBCPs	0.1	5.71 (4.77 - 6.49)	38.43 (36.2 - 41.29)
	Female	BMI+WHR	0.03	2.92 (2.31 - 3.63)	60.82 (58.04 - 64.07)
		BMI+WHR+adjBCPs	0.03	3.15 (2.49 - 3.75)	61.24 (57.58 - 64.88)
		BMI+WHR	0.04	1.62 (1.16 - 2.14)	26.17 (24.69 - 29.24)
		BMI+WHR+adjBCPs	0.04	1.81 (1.32 - 2.37)	28.17 (26.28 - 30.57)
		BMI+WHR	0.05	0.93 (0.58 - 1.35)	11.72 (10.39 - 13.48)
		BMI+WHR+adjBCPs	0.05	0.97 (0.57 - 1.38)	14.27 (12.55 - 15.67)
		BMI+WHR	0.1	0 (0 - 0)	0.51 (0.19 - 0.74)
		BMI+WHR+adjBCPs	0.1	0.05 (0 - 0.14)	0.83 (0.37 - 1.25)
Diabetes	Male	BMI+WHR	0.05	43.25 (41.67 - 44.49)	330.47 (324.88 - 336.54)
		BMI+WHR+adjBCPs	0.05	43.63 (42.48 - 44.76)	305.82 (300.52 - 311.74)
		BMI+WHR	0.1	29.26 (27.72 - 30.63)	135.26 (131.49 - 139.7)
		BMI+WHR+adjBCPs	0.1	30.06 (28.86 - 31.54)	128.28 (123.92 - 132.59)
		BMI+WHR	0.15	19.03 (17.25 - 20.25)	62.58 (60 - 65.91)
		BMI+WHR+adjBCPs	0.15	21.99 (20.83 - 23.7)	61.27 (58.12 - 64.23)
		BMI+WHR	0.2	11.29 (10.18 - 12.32)	27.95 (26.02 - 29.85)
		BMI+WHR+adjBCPs	0.2	15.64 (14.13 - 16.91)	32.9 (30.68 - 34.97)
	Female	BMI+WHR	0.05	24.88 (23.59 - 26.28)	216.89 (209.94 - 221.33)
		BMI+WHR+adjBCPs	0.05	24.27 (22.99 - 25.45)	169.82 (164.47 - 175.89)
		BMI+WHR	0.1	15.01 (13.56 - 16.42)	72.4 (69.03 - 76.09)
		BMI+WHR+adjBCPs	0.1	16.91 (15.53 - 18.07)	62.17 (58.9 - 66.1)
		BMI+WHR	0.15	8.43 (7.36 - 9.48)	27.84 (25.68 - 29.89)
		BMI+WHR+adjBCPs	0.15	11.49 (10.1 - 12.57)	32.66 (30.62 - 35.12)
		BMI+WHR	0.2	4.96 (4.15 - 5.78)	9.87 (8.52 - 10.96)
		BMI+WHR+adjBCPs	0.2	8.8 (7.69 - 10.07)	18.9 (17.36 - 20.4)
Hyper-lipidemia	Male	BMI+WHR	0.5	294.49 (290.19 - 298.94)	184.01 (180.37 - 188.38)
		BMI+WHR+adjBCPs	0.5	292.55 (288.1 - 296.24)	170.82 (166.85 - 175.84)
		BMI+WHR	0.6	163.16 (159.89 - 167)	90.91 (88.04 - 94.3)
		BMI+WHR+adjBCPs	0.6	177.96 (173.53 - 181.87)	89.72 (85.87 - 94.04)
	Female	BMI+WHR	0.5	205.36 (201.31 - 209.83)	126.28 (121.1 - 130.9)
		BMI+WHR+adjBCPs	0.5	205.86 (201.26 - 209.99)	120.54 (116.44 - 124.98)
		BMI+WHR	0.6	110.07 (106.07 - 114.24)	58.88 (56.23 - 62.17)
		BMI+WHR+adjBCPs	0.6	116.88 (113.19 - 120.8)	57.16 (54.01 - 60.35)


Supplementary Figure 1. Participant inclusion flowchart. From an initial sample of 30,397 NAKO and 19,512 UKB MRI scans, participants were sequentially filtered: after confirming required imaging modalities and image sizes (yielding 30,039 NAKO, 19,421 UKB), excluding scans with fat fraction ≥ 0.6 in larger areas (29,902 NAKO, 19,164 UKB), and retaining those with height, weight, and clinical condition status data (29,114 NAKO, 19,164 UKB), the final analytic cohorts comprised 26,877 NAKO and 18,974 UKB participants with available health behaviour information.


Supplementary Figure 2. Baseline characteristics and body composition parameters. (a) Histograms and density plots of participants baseline characteristics stratified by dataset and sex. (b) Histograms illustrate the distributions of ASAT, GFAT, VAT, trunk muscle volume, and LFF from NAKO and UKB cohorts, stratified by dataset and sex, before (top row) and after (bottom row) applying ComBat harmonization. This adjustment minimizes site-specific differences while preserving biological variation. (c) Charts display the contributions of individual muscles form the core, hip, and lower back region to total trunk muscle volume, stratified by dataset and sex. The left panels show the raw values, while the right panels display the data after ComBat harmonization. (d) Distributions of BCPs by BMI category for men and women, displayed as histograms with dashed lines indicating medians. Pearson correlation coefficients between each BCP and BMI are shown in the top row.


Supplementary Figure 3. Estimated associations between health behaviour indicators and unadjusted BCP Z-scores, derived from linear mixed-effects models stratified by cohort and adjusted for age, height, BMI, and available behavioural covariates (hand grip strength and physical activity in NAKO; smoking and alcohol intake in UKB). Imaging centre was included as a random intercept. All associations reflect cross-sectional patterns and are not indicative of causal relationships. For detailed model statistics see Supplementary Tables 4 and 5.


Supplementary Figure 4. Distribution and correlation of body composition parameter residuals after adjustment for anthropometric covariates. (a) Histograms illustrate the distribution of model residuals for abdominal subcutaneous adipose tissue (ASAT), gluteofemoral adipose tissue (GFAT), visceral adipose tissue (VAT), trunk muscle volume, and liver fat fraction (LFF), stratified by sex and BMI category. All measures show approximately normal distributions with minimal systematic bias across BMI categories, validating our statistical approach for isolating depot-specific adiposity measures independent of overall body size. (b) LOESS curves display the mean and ±1 SD for height-adjusted (except for LFF) and BMI-adjusted body composition parameters across age. Black lines indicate the mean, and shaded areas represent ±1 SD. (c) Correlation matrix of adjusted BCPs (residuals after adjustment for age, height, and BMI), with Pearson coefficients presented for men (upper triangle) and women (lower triangle); all correlations p < 0.0001 from two-sided t-tests. Pearson correlation coefficients with 95% CI can be found in Supplementary Table 6.


Supplementary Figure 5. Odds ratios based on adjusted body-composition parameters stratified by sex. Forest plots show cross-sectional associations between adjusted body-composition parameters and clinical conditions, stratified by sex. (a) ASAT-adj showed modest positive associations with hyperlipidemia and diabetes, particularly in males. (b) GFAT-adj showed inverse associations with metabolic conditions, including lower odds of diabetes (OR = 0.66–0.71, P < 0.0001) and hyperlipidemia (OR = 0.86–0.88, P < 0.0001). (c) VAT-adj showed the strongest positive associations with cardiovascular and metabolic conditions in both sexes, with a female-specific association with cancer (OR = 1.12, P = 0.0012). (d) Adjusted trunk-muscle volume showed stronger inverse associations in men, including lower odds of heart failure (OR = 0.81, P = 0.0076), diabetes (OR = 0.79, P < 0.0001), and osteoporosis (OR = 0.73, P < 0.001). (e) LFF-adj was positively associated with metabolic conditions, particularly diabetes (OR = 1.26–1.32, P < 0.0001). These results describe sex-specific cross-sectional patterns in how regional adiposity and trunk-muscle volume relate to prevalent cardiometabolic, cancer, and related clinical conditions independent of BMI.


Supplementary Figure 6. Sex-separated body-composition phenotypes by clinical-condition status. Body-composition phenotypes are presented as mean Z-scores ± standard error of the adjusted body-composition parameters (BCPs). The Healthy reference comprises UKB participants only because its definition required absence of all nine conditions, including cancer, which was unavailable in NAKO; non-cancer condition groups otherwise pool both cohorts. The top panel shows cardiovascular conditions (CAD, heart failure, hypertension), the middle panel metabolic disorders (diabetes, gout, hyperlipidemia), and the bottom panel other conditions (back pain, cancer, osteoporosis). These descriptive profiles show condition- and sex-related differences; they do not constitute formal cohort-equivalence tests.


Supplementary Figure 7. Predicted prevalence plots of other clinical conditions. Plots show the predicted prevalence with confidence intervals of the remaining available clinical conditions (cancer, heart failure, gout, osteoporosis, and back pain) across Z-scores of adjusted body composition parameters (BCPs) based on generalized mixed-effects logistic regression models adjusted for age, BMI, adjusted BCPs, and interaction terms between BMI and each of the BCPs, with imaging centre as a random effect. Data are stratified by sex and BMI category (set at representative values of 20 kg/m2 (normal), 27.5 kg/m2 (overweight), and 35 kg/m2 (obese)).


Supplementary Figure 8. Extraction of waist and hip circumference. The hip measurement plane was defined as the midpoint between the left and right femoral heads and the iliac bones. For the waist, the measurement plane was defined at the level of the third lumbar vertebra (L3). To simulate tape-based circumference measurements, we calculated perimeters on axial slices at these predefined locations using merged subcutaneous and gluteofemoral adipose tissue. To ensure a continuous enclosing contour, we applied morphological closing to fill small discontinuities, followed by a convex hull operation to bridge any remaining gaps. To validate our method, we compared MRI-derived with manually measured waist and hip circumferences in NAKO participants (n = 3,726 available). MRI-derived values consistently overestimated manual measures (hip: +4.8 cm, waist: +7.8 cm) across 3,572 (hip) and 3,581 (waist) out of the 3,726 individuals.  Agreement with manual measures was high (r = 0.94, p < 0.001) for both waist and hip circumferences.


Supplementary Figure 9. Receiver operating characteristic (ROC) curves from generalized linear mixed-effects models of other outcomes comparing performance for males and females separately. Shown are base models (including age only), models with added anthropometric variables (BMI and waist-to-hip ratio), and models further including adjusted body composition parameters (adjusted BCPs).


Supplementary Figure 10.  Calibration curves for models of all outcomes. Dots represent deciles of predicted probability. For intercept and slope values, see Supplementary Tab. 10.


Supplementary Figure 11. Decision curve analysis with plots showing standardized net benefit across a range of probability thresholds as well as “treat all” and “treat none” strategies for models of CAD, type 2 diabetes, and hyperlipidemia. Higher net benefit indicates greater clinical utility for guiding clinical decisions. Metrics of true and false positives at select probability thresholds can be found in Supplementary Tab. 11. 


Supplementary Table 12. Two-reader segmentation agreement on a 50-scan development-sequence subset.
Two board-certified radiologists independently segmented a 50-scan subset drawn from UKB Dixon and NAKO T2-HASTE images, blinded to model output and to each other. Reader–reader Dice and algorithm–reader Dice were summarized by compartment; algorithm–reader Dice is the mean of model-versus-reader 1 and model-versus-reader 2. Volume bias and 95% limits of agreement were calculated by Bland–Altman analysis, with reader–reader bias defined as reader 1 minus reader 2 and algorithm–reader bias as model minus the reader mean. Only aggregate statistics are reported. This analysis contextualizes agreement on the annotated development-sequence domains and does not validate the NAKO GRE population input.
Compartment	Reader–reader Dice, mean (95% CI)	Algorithm–reader Dice, mean (95% CI)	Reader–reader volume bias (95% LoA), ml	Algorithm–reader volume bias (95% LoA), ml
SAT	0.953 (0.947–0.959)	0.924 (0.918–0.931)	+232 (-1004 to +1468)	-32 (-677 to +614)
ASAT	0.946 (0.941–0.951)	0.917 (0.911–0.923)	-66 (-725 to +593)	+61 (-276 to +399)
GFAT	0.936 (0.930–0.943)	0.904 (0.898–0.909)	-44 (-935 to +848)	-59 (-449 to +330)
VAT	0.898 (0.893–0.904)	0.865 (0.860–0.870)	-12 (-692 to +668)	+174 (-436 to +785)
Trunk muscle	0.951 (0.945–0.956)	0.932 (0.926–0.938)	+34 (-420 to +489)	-33 (-387 to +322)
Overall (all compartments)	0.937 (0.933–0.940)	0.908 (0.905–0.912)	—	—


Supplementary Figure 12. Bland–Altman analysis of reader–reader and algorithm–reader volume agreement in the 50-scan development-sequence subset.
Difference versus mean of segmented compartment volumes (ml). Green circles show reader 1 minus reader 2; orange triangles show model minus the reader mean. Solid lines denote mean bias and dashed lines the 95% limits of agreement. Scans were drawn from UKB Dixon and NAKO T2-HASTE development-sequence images; no scan identifiers or participant characteristics are shown. This figure does not assess the NAKO GRE population input.
Supplementary References
1. Gatidis S, Kart T, Fischer M, Winzeck S, Glocker B, Bai W, Bülow R, Emmel C, Friedrich L, Kauczor H-U. Better together: data harmonization and cross-study analysis of abdominal MRI data from UK Biobank and the German National Cohort. Investigative radiology 2023;58(5):346-354. 
2. Johnson WE, Li C, Rabinovic A. Adjusting batch effects in microarray expression data using empirical Bayes methods. Biostatistics 2007;8(1):118-127. 
3. Joo I, Kwak M-S, Park DH, Yoon SH. Fully automated waist circumference measurement on abdominal CT: Comparison with manual measurements and potential value for identifying overweight and obesity as an adjunct output of CT scan. PLoS One 2021;16(7):e0254704.
4. Kofler, F. et al. Robust, primitive, and unsupervised quality estimation for segmentation ensembles. Front. Neurosci. 15, 752780 (2021). https://doi.org/10.3389/fnins.2021.752780.
